# Parallel evolution of arborescent carrots (*Daucus*) in Macaronesia

**DOI:** 10.1002/ajb2.1444

**Published:** 2020-03-08

**Authors:** Kamil E. Frankiewicz, Alexei Oskolski, Łukasz Banasiak, Francisco Fernandes, Jean‐Pierre Reduron, Jorge‐Alfredo Reyes‐Betancort, Liliana Szczeparska, Mohammed Alsarraf, Jakub Baczyński, Krzysztof Spalik

**Affiliations:** ^1^ Department of Molecular Phylogenetics and Evolution Institute of Botany Faculty of Biology University of Warsaw Biological and Chemical Research Centre Żwirki i Wigury 101 02‐089 Warsaw Poland; ^2^ Department of Botany and Plant Biotechnology University of Johannesburg PO Box 524, Auckland Park 2006 Johannesburg South Africa; ^3^ Botanical Museum Komarov Botanical Institute Prof. Popov 2 197376 St. Petersburg Russia; ^4^ Instituto das Florestas e Conservação da Natureza Quinta Vila Passos, R. Alferes Veiga Pestana 15 9054‐505 Funchal, Madeira Portugal; ^5^ Via Apia, 10 rue de l'Arsenal 68100 Mulhouse France; ^6^ Jardín de Aclimatación de la Orotava (ICIA) C/Retama no. 2 38400 Puerto de la Cruz, S/C de Tenerife Spain

**Keywords:** Apiaceae, Daucinae, habit evolution, insular woodiness, *Melanoselinum*, molecular dating, *Monizia*, secondary woodiness, *Tornabenea*, wood anatomy

## Abstract

**Premise:**

Despite intensive research, the pathways and driving forces behind the evolution of derived woodiness on oceanic islands remain obscure. The genus *Daucus* comprises mostly herbs (therophytes, hemicryptophytes) with few rosette treelets (chamaephytes) endemic to various Macaronesian archipelagos, suggesting their independent evolution. To elucidate the evolutionary pathways to derived woodiness, we examined phylogenetic relationships and the habit and secondary xylem evolution in *Daucus* and related taxa.

**Methods:**

Sixty taxa were surveyed for molecular markers, life history, and habit traits. Twenty‐one species were considered for wood anatomical characters. A dated phylogeny was estimated using Bayesian methods. The evolution of selected traits was reconstructed using parsimony and maximum likelihood.

**Results:**

*Daucus* dispersed independently to the Canary Islands (and subsequently to Madeira), Cape Verde, and the Azores in the late Miocene and Pleistocene. Life span, reproductive strategy, and life form were highly homoplastic; the ancestor of *Daucus* was probably a monocarpic, biennial hemicryptophyte. Rosette treelets evolved independently in the Canarian‐Madeiran lineage and in Cape Verde, the latter within the last 0.13 Myr. Treelets and hemicryptophytes did not differ in wood anatomy. Pervasive axial parenchyma in wood occurred more often in polycarpic rather than monocarpic species.

**Conclusions:**

Life span and life form in *Daucus* are evolutionarily labile and may change independently of wood anatomy, which is related to plant reproductive strategy rather than to life form. Insular woodiness may evolve rapidly (as demonstrated in *D. bischoffii*), and in *Daucus*, it does not seem to be an adaptation to lower the risk of xylem embolism.

Woodiness was most likely ancestral within angiosperms, and the shift from trees and shrubs to herbs has long been considered a major trend in the evolution of flowering plants (e.g., Sinnott and Bailey, 1915); however, a large body of evidence suggests that the opposite is also possible and that many woody taxa in various angiosperm lineages originated from herbaceous ancestors (reviewed, e.g., by Dulin and Kirchoff, 2010). Phenomenon of such evolutionary reversal, known as secondary or (phylogenetically) derived woodiness, is often associated with insular woodiness, i.e., the tendency of herbaceous plants to evolve into rosette trees, shrubs, and other arborescent life forms after dispersal to islands. Both phenomena, and especially insular woodiness, have been extensively discussed since the 19th century, and numerous hypotheses have been proposed to explain them (e.g., Darwin, 1859; Wallace, 1878; Carlquist, 1974). Darwin suggested increased competition between herbaceous species favoring taller individuals; Carlquist (1974) postulated that moderate climates with low seasonality and/or the absence of large herbivores may be responsible for the shift; and recently, Lens et al. (2013b) and Dória et al. (2018) considered drought adaptation to be the trigger because derived woody stems have higher embolism resistance than herbaceous relatives. Each of those hypotheses has its limitations; moreover, they are not mutually exclusive. To the contrary, a single causal scenario explaining all cases of derived woodiness is unlikely (Kidner et al., 2015; Carlquist, 2017). Therefore, to explain the evolution of insular woodiness more synthetic studies combining anatomical and ecological approaches with robust phylogenetic background are needed.

Attempts to discover anatomical features that distinguish derived woody plants from ancestral woody ones led to the theory of paedomorphosis in wood evolution (Carlquist, 1962), which has been extensively studied (Lens et al., 2005, 2007, 2009, 2012a; Neupane et al., 2017) and reviewed in recent years (Carlquist, 2009a, b, 2012, 2013; Dulin and Kirchoff, 2010; Lens et al., 2013a). According to this theory, juvenile or “paedomorphic” traits are expected to persist in the mature wood of derived woody plants. However, Lens et al. (2013a) argued that at least some traits usually interpreted as being indicators of protracted juvenilism result instead from some distinctive growth forms (rosette trees, stem succulents, and slender‐stem shrubs) irrespective of their derived or ancestral woodiness. Also, several wood traits not necessarily interpreted as juvenile are known to correlate with plant habit (e.g., Carlquist, 1984; Carlquist and Donald, 1996; Carlquist and DeVore, 1998; Olson and Carlquist, 2001; Carlquist and Grant, 2006; Arévalo et al., 2017). Carlquist incorporated this view in his later works, placing the emphasis of the explanation of protracted juvenilism not only with plant evolutionary history, but also with plant growth form (Carlquist, 2009b) and trait function (Carlquist, 2012, 2015a, b, 2018).

Experimental data on the model plant *Arabidopsis thaliana* suggest that the gain of a prominent wood cylinder requires small genetic changes (Chaffey et al., 2002; Ko et al., 2004; Melzer et al., 2008; Lens et al., 2012b; Rowe and Paul‐Victor, 2012; Davin et al., 2016); therefore, derived woodiness may evolve in a relatively short time. However, even though derived woodiness is a widespread phenomenon found in many families (Park et al., 2001; Barber et al., 2002; Francisco‐Ortega et al., 2002; Lee et al., 2005; Nürk et al., 2019), the ages of derived and, in particular, insular woody clades remain mostly unknown (Kim et al., 2008), obstructing our understanding of the possible causes and mechanisms leading to the evolution of derived woodiness.

Subtribe Daucinae (Apiaceae, Apioideae) is a promising group for testing the hypotheses explaining the relationships between habit shifts, wood trait evolution, and insular diversification. The tribe comprises some 93 species distributed mostly around the Mediterranean, in Europe, western Asia, tropical Africa and in Macaronesia, of which the cultivated carrot (*Daucus carota*; authorities are given in Appendix S1) is the best‐known representative (Banasiak et al., 2013, 2016). Most species are herbaceous; however, there are a few exceptions characterized by substantial height and deposition of a significant amount of secondary xylem (Fig. 1). The most impressive examples are *Daucus decipiens* (≡ *Melanoselinum decipiens*) and its sister species *Daucus edulis* (≡ *Monizia edulis*), both rosette trees from Madeira reaching 4 m in height (Lowe, 1923; Fernandes and Carvalho, 2014). Stems with a prominent woody base of up to 0.5 m in height are also characteristic for the biennial *Daucus elegans* (≡ *Cryptotaenia elegans*) from the Canary Islands. In addition, the formation of a slender woody stem up to 1 m tall has been reported by Brochmann et al. (1997) for *Daucus bischoffii* (≡ *Tornabenea bischoffii*) and *Daucus tenuissimus* (≡ *Tornabenea tenuissima*) from Cape Verde. However, these two species are also considered to be herbaceous (Martins, 1996).

**Figure 1:**
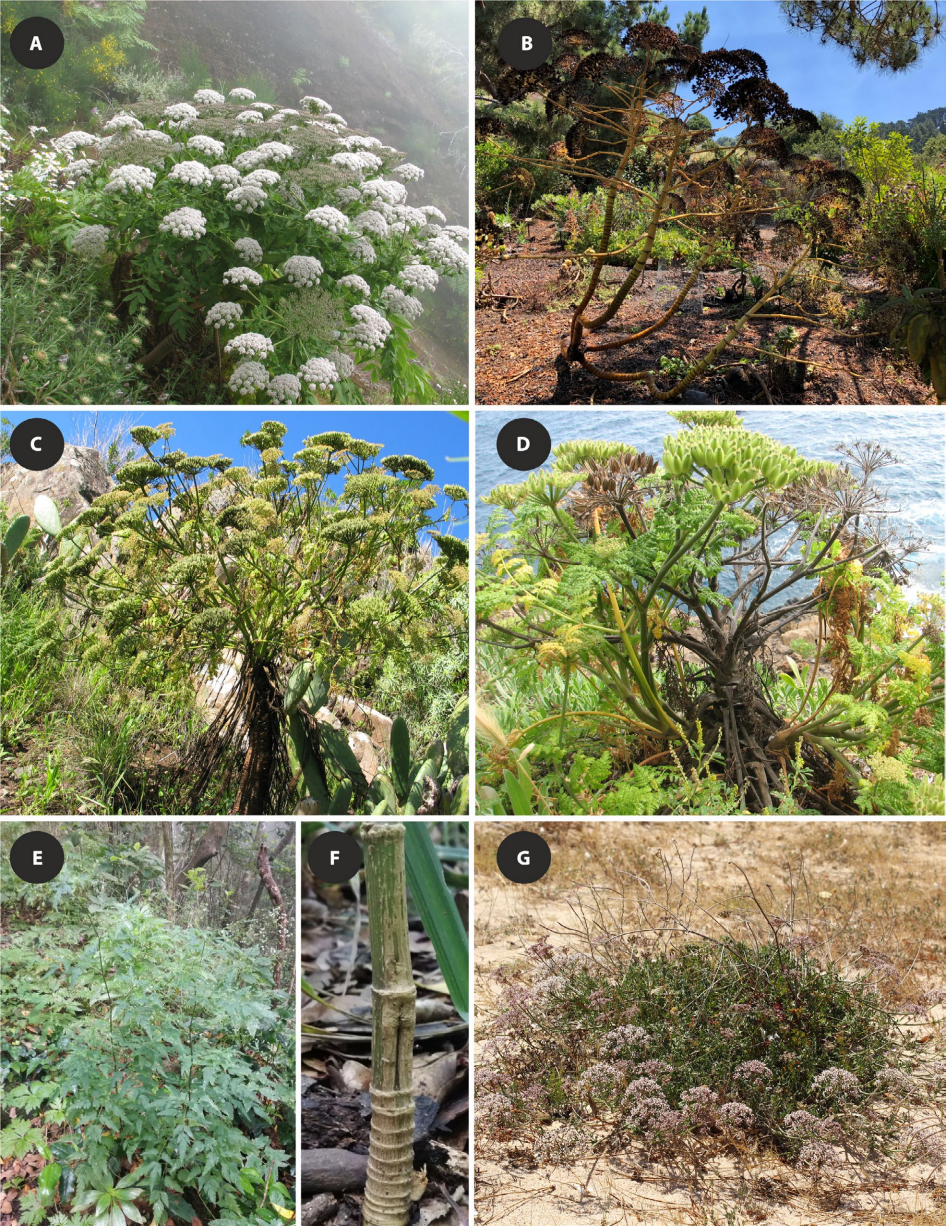
Life form and life history variation among exemplar species under study. Monocarpic perennial *Daucus decipiens* (A) before and (B) after fruiting. Polycarpic perennial *Daucus edulis* with (C) prominent stem and (D) remains of previous‐year umbels. (E) Monocarpic biennial *Daucus elegans* with (F) woody basal part of stem. (G) Polycarpic perennial *Daucus rouyi*; only upper parts of this sand dune plant are visible.

Although shrubs and trees are uncommon in Apiaceae subfamily Apioideae, some authors suggest that the woody habit—considered as shrubby or arborescent life form and, simultaneously, deposition of secondary xylem—is plesiomorphic for the subfamily because its basal lineages are predominantly woody (Oskolski, 2001; Oskolski and Van Wyk, 2008; Stepanova and Oskolski, 2010; Long and Oskolski, 2018). However, formal ancestral state reconstruction inferred herbaceousness for the most recent common ancestor of this clade (Winter et al., 2008; Magee et al., 2009, 2010). Regardless of the ancestral condition for subfamily Apioideae, the woody members of Daucinae, as one of its crown groups, are definitely derived from herbaceous ancestors, and this character has been shown to have a substantial level of homoplasy in various clades of the subfamily (Calviño et al., 2006; Oskolski and Van Wyk, 2008; Downie et al., 2010; Magee et al., 2010; Banasiak et al., 2016).

Recent phylogenetic and taxonomic studies of Daucinae have shown that various woody species that had been previously scattered among the genera *Athamanta*,* Cryptotaenia*,* Melanoselinum*,* Monizia*,* Rouya*, and *Tornabenea* are nested within *Daucus* s.l. and have been transferred to the latter (Banasiak et al., 2016). The former monotypic genera *Melanoselinum* and *Monizia* form a clade (*Daucus* sect. *Melanoselinum*) closely related to *Daucus* s.s. (≡ sect. *Daucus*, which includes the generitype, *D. carota*), whereas the studied representatives of *Tornabenea*, as well as *Daucus rouyi* (≡ *Rouya polygama*) and *Daucus della‐cellae* (≡ *Athamanta della‐cellae*), are nested within sect. *Daucus*. However, the phylogenetic positions of *D. elegans* from the Canary Islands and *D. bischoffii* (≡ *T. bischoffii*), a rosette treelet from Cape Verde, remain obscure. The former was resolved as either the sister to the two Madeiran *Daucus* species (*D. decipiens* and *D. edulis*; Spalik and Downie, 2007; Banasiak et al., 2013) or included in sect. *Daucus* as the sister to the remaining species of sect. *Daucus* (Banasiak et al., 2016). The phylogenetic position of *D. bischoffii* has never been investigated with molecular data. It is possible that *D. bischoffii* is nested within sect. *Daucus*, like other former members of *Tornabenea*. However, as in the case of *D. elegans*, it is also possible that the species is related to the Madeiran endemics forming sect. *Melanoselinum* (Fig. 2), a scenario already considered by Chevalier (1935).

**Figure 2:**
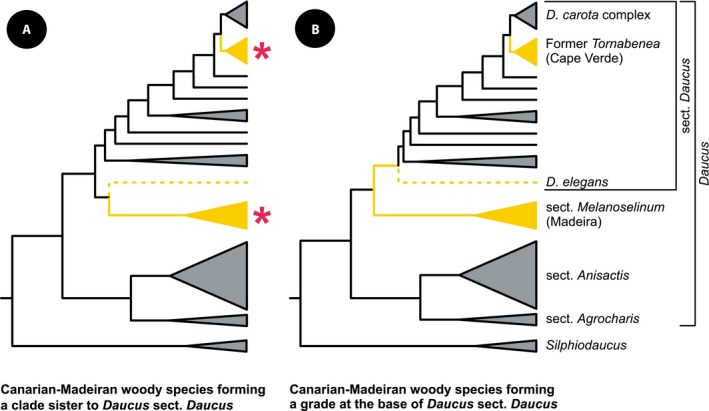
Phylogenetic hypotheses on the relationships among insular treelets in *Daucus*. In (A), *Daucus elegans* (marked in dotted line) is sister to a clade of Madeiran endemics (*Daucus decipiens* and *Daucus edulis*, i.e., *Daucus* sect. *Melanoselinum*). This scenario was reconstructed by Spalik and Downie (2007) and Banasiak et al. (2013). In (B), *D. elegans* is sister to the remaining species of *Daucus* sect. *Daucus*, scenario inferred by Banasiak et al. (2016). Clades comprising woody species are marked in yellow. Asterisks denote hypothetical alternative positions of *Daucus bischoffii*.

Resolving the phylogenetic positions of *D. elegans* and *D. bischoffii* is crucial for understanding life‐form evolution in sect. *Daucus* and the evolution of wood traits in Daucinae. If *D. elegans* and *D. bischoffii* are the closest relatives of the species from Madeira and Cape Verde, respectively, then the ancestor of sect. *Daucus* was most likely herbaceous, while the chamaephytic, or treelet, life form probably evolved independently in these two Macaronesian clades (Fig. 2A). However, if *D. elegans* is sister to the remaining species of sect. *Daucus*, then the Macaronesian treelets form a grade at the base of the group and the treelet life form is probably ancestral for sect. *Daucus* (Fig. 2B). This scenario implies a loss of the chamaephytic life form early in the evolution of sect. *Daucus* and its subsequent regain in the lineage of species formerly placed in *Tornabenea*.

It should be kept in mind that woodiness and herbaceousness are highly ambiguous concepts that can refer either to habit or to stem anatomy. The term *woodiness* can be used to describe both plants having prominent perennial aboveground stems, as well as the ones producing a distinct wood cylinder that extends toward upper stem parts (Kidner et al., 2015). Correspondingly, a plant can be recognized as herbaceous on the basis of either a life history in which aboveground parts die off at the end of the growing season or the absence or limited amount of secondary growth in stems. These two approaches to the definition of woodiness and herbaceousness are not necessarily correlated with each other; there are many examples of plants having a typical tree habit without secondary growth and of annual herbs with normal wood cylinders (Dulin and Kirchoff, 2010; Schweingruber and Büntgen, 2013). The question whether wood evolution follows evolutionary change in life form remains unanswered in Daucinae. It might turn out that both traits are highly correlated—hemicryptophytes being distinguishable from their chamaephytic/rosette treelet relatives based on wood anatomical traits—but it is equally likely that both groups share similar xylem features. So far almost all anatomically studied Apioideae species have been shown to deposit some amount of secondary xylem (e.g., Oskolski, 2001; Oskolski and Van Wyk, 2008; Schweingruber and Landolt, 2010; Stepanova and Oskolski, 2010; Long and Oskolski, 2018; our unpublished data), and for this reason, technically they all could be called woody. However, to identify the most dramatic shifts in the amount of wood deposition within Daucinae, we consider species with limited secondary growth, mostly therophytes and hemicryptophytes, as herbaceous, whereas as woody we describe only those with prominent secondary xylem, i.e., chamaephytes and rosette treelets. Simultaneously, we consider the evolution of habit and wood anatomical *traits* separately using Raunkiaer (1934) terms to characterize the life forms of species irrespective of wood anatomical structure.

Our study had two main objectives. First, to resolve the phylogenetic positions of *D. elegans* and *D. bischoffii* and to estimate the timescale of the Daucinae radiation to reconstruct the evolution of life form in this group and particularly to estimate the timing of the origin(s) of insular treelets. Second, we investigate wood anatomy of selected species (including all insular woody ones) to determine whether there are any wood traits that distinguish derived woody species of *Daucus* from their herbaceous relatives.

## MATERIALS AND METHODS

### Molecular analyses

Based on a previous study (Banasiak et al., 2016), we selected 60 species and subspecies representing all major lineages of subtribe Daucinae and three species representing other subtribes of Scandiceae as an outgroup. Material obtained from herbaria or living collections was used to isolate total DNA from ca. 20 mg of dried leaves using DNeasy Plant Mini Kit (Qiagen, Venlo, Netherlands). Four of the molecular markers used in this study—nuclear ribosomal DNA internal transcribed spacer (nrDNA ITS) and plastid *rpoC1* and *rps16* introns and *rpoB‐trnC* intergenic spacer—have previously been used in phylogenetic analyses of the subtribe (Banasiak et al., 2016). Additionally, we analyzed sequence variation of nuclear ribosomal DNA external transcribed spacer (nrDNA ETS) and plastid *rpl16* intron. Loci were amplified by PCR using previously developed protocols and primers (Logacheva et al., 2010; Banasiak et al., 2016). Sanger sequencing was performed by Genomed S.A. (Warsaw, Mazovia, Poland), and obtained reads were assembled using SeqMan Pro 13.0.2 (DNAStar, Madison, WI, USA). Newly obtained sequences were deposited in GenBank (Appendix S1).

The sequences were aligned using the E‐INS‐i algorithm implemented in MAFFT 7.271 (Katoh and Standley, 2013). Primer and partial exon sequences flanking the noncoding regions were manually trimmed in Mesquite 3.51 (Maddison and Maddison, 2018). Subsequently, the automated1 algorithm implemented in trimAl 1.2 (Capella‐Gutiérrez et al., 2009) was used to remove ambiguously aligned positions.

### Phylogeny estimation and node calibration

**Figure 3:**
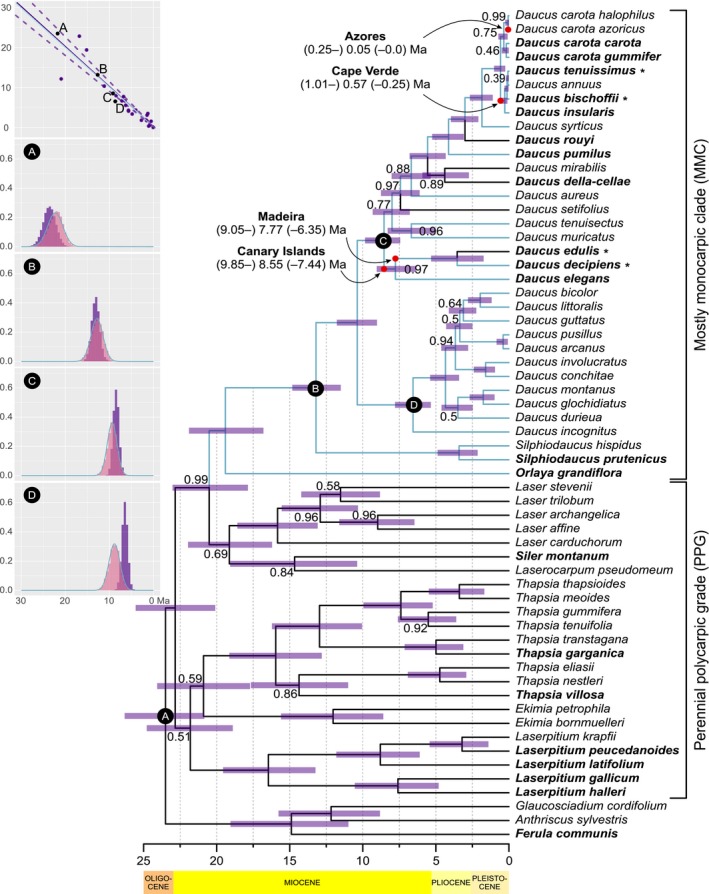
Maximum clade credibility tree summarizing Bayesian MCMC analyses of Daucinae and outgroups with BEAST. Scatter plot shows correlation of median ages for corresponding nodes in primary (abscissa axis) and secondary (ordinate axis) calibrated tree. Solid and dashed violet lines represent major axis with 95% confidence interval compared to blue line expected under assumption of no systematic bias in age estimates. A, B, C, and D denote calibration points with respective plots of prior (blue curves) and posterior (violet histograms) age distributions. Pink histograms represent posterior age distributions for the calibration points from previous study (Banasiak et al., 2013), which were used to calculate parameters for priors. Violet bars represent 95% highest posterior density intervals for age distributions. Red dots mark possible dispersals to and within Macaronesia. Branches in black were reconstructed with maximum parsimony as polycarpic, while those in blue as monocarpic. For clarity, only posterior probability values lower than 1.0 are shown. Taxa included in the anatomical study are marked in boldface; insular rosette treelets are marked with asterisks, and time scale is in millions of years ago.

The BIC metric was used to choose the optimal partitioning scheme and nucleotide substitution models in PartitionFinder 2 (Lanfear et al., 2012). The substitution models were confined to those available in BEAST 1.10.0 (Drummond and Rambaut, 2007). The congruence of phylogenetic signal between nuclear and plastid molecular data sets was assessed using hierarchical likelihood ratio test implemented in Concaterpillar 1.7.2. (Leigh et al., 2008). Topology estimation was performed simultaneously with molecular dating. Since Daucinae lack a reliable fossil record, we used four secondary calibration points based on a Bayesian dating of subfamily Apioideae (Banasiak et al., 2013). All chosen nodes had a posterior probability of 1.0 in the analyses of Banasiak et al. (2013) and represented the most recent common ancestors of tribe Scandiceae (Fig. 3A), *Daucus* and *Silphiodaucus* (B), *Daucus* sect. *Daucus* and sect. *Melanoselinum* (C), *Daucus* sect. *Agrocharis* and sect. *Anisactis* (D). For each of these nodes, the age posterior distribution was evaluated based on a sample of 10,800 phylogenetic trees from Banasiak et al. (2013) using treeStat 1.10.0 (Rambaut and Drummond, 2018b). Because the posterior distributions did not considerably deviate from the normal distribution as assessed by normal quantile‐quantile plots in R (R Core Team, 2008), the calibration points were defined as having normal distributions with mean and standard deviation calculated based on the respective posterior samples. We used the random local clock model to accommodate differences in the rate of molecular evolution between lineages representing annual, biennial, and perennial taxa. Two independent Markov chains were run for 100,000,000 generations each and sampled every 10,000 generations. The initial 20% of trees from each run were discarded as burn‐in. Posterior distributions of parameter estimates were inspected in Tracer 1.7.1 (Rambaut et al., 2018). Both runs converged on the same stationary distribution and were combined for subsequent analyses using LogCombiner 1.10.0 (Rambaut and Drummond, 2018a). The resulting set of 16,000 trees was summarized using TreeAnnotator (Drummond and Rambaut, 2007) in a maximum clade credibility (MCC) tree. For diagnostic purposes, the posterior distributions of ages for nodes from the Banasiak et al. (2013) serving as secondary calibration points were plotted against the results of current study using the ggplot (Wickham, 2009) package in R. Additionally, we compared median ages for all pairs of corresponding nodes between primary (Banasiak et al., 2013) and secondary calibration (this study) fitting major axis with fixed zero intercept, i.e., optimizing only for slope parameter, using R package smatr (Warton et al., 2012).

### Wood anatomical analyses

Twenty‐one species were considered for wood anatomical characters: data for 10 species were gathered from the literature (Schweingruber and Landolt, 2010), while 11 species were newly examined for this study. These newly examined species were represented by 15 specimens obtained from herbaria (E, FR, WA), living collections (Conservatoire Botanique de Mulhouse, France and Jardim Botânico da Madeira, Portugal), and wild populations (Tenerife, Anaga Mountains, Vueltas de Taganana). We tried to cover all species described as woody in the literature, regardless of reference to plant habit or anatomy. We included all cases of presumed insular woodiness (i.e., insular rosette treelets: *D. decipiens*,* D. edulis*,* D. bischoffii*,* D. tenuissimus*; and an insular chamaephyte with prominent woody base: *D. elegans*) and nearly all other species in the subtribe for which various degrees of woodiness were reported (for example, Lowe, 1923; Brochmann et al., 1997; Table 1). Voucher specimens are listed in Appendix S1. Wood samples were taken from the basalmost part of the stem in the generative phase. Exceptionally, *Daucus decipiens* and *D. edulis* were sampled at both generative and vegetative phases from underground, basal, middle, and apical parts of the stem. Fresh material was immediately stored in 70% ethanol.

**Table 1:** Selected distribution and life span, reproductive strategy and habit traits of Daucinae species examined for anatomical characters. Slashes separate polymorphisms; asterisks indicate the dominant state.

Species	Insular woodiness	Life span	Reproductive strategy	Shoot system	
				Aboveground	Underground
*Daucus bischoffii*	Yes (Cape Verde)	Annual/perennial*	Monocarpic	Annual monocarpic stems with or without rosette	Long unbranched rhizome
*Daucus carota*	No	Annual/biennial*/perennial	Monocarpic	Annual monocarpic stem with basal rosette	None or unbranched short rhizome
*Daucus decipiens*	Yes (Madeira)	Biennial/perennial*	Monocarpic	Perennial monocarpic stem with terminal rosette	None
*Daucus della‐cellae*	No	Perennial	Polycarpic	Annual monocarpic stems with basal rosette	Short or long branched rhizome
*Daucus edulis*	Yes (Madeira)	Perennial	Polycarpic	Perennial polycarpic stem with terminal rosette	None
*Daucus elegans*	Yes (Canary Islands)	Annual/biennial*/perennial	Monocarpic	Perennial monocarpic stem without terminal rosette	None
*Daucus insularis*	Yes (Cape Verde)	Annual/perennial	Monocarpic	Annual monocarpic stems without rosette	Long unbranched rhizome
*Daucus rouyi*	Doubtful (Corsica, N Africa)	Perennial	Polycarpic	Perennial monocarpic stems without rosette	Long branched rhizome
*Daucus tenuissimus*	Yes (Cape Verde)	Annual/perennial	Monocarpic	Annual monocarpic stems without rosette	Short unbranched rhizome or none
*Laserpitium latifolium*	No	Perennial	Polycarpic	Annual monocarpic stem with basal rosette	Short unbranched rhizome
*Silphiodaucus prutenicus*	No	Biennial	Monocarpic	Annual monocarpic stem with basal rosette	Short unbranched rhizome

Wood samples were soaked in boiling water until they sank. They were then sectioned using a sledge microtome (SM2010R, Leica Biosystems, Wetzlar, Hesse, Germany) resulting in 20–60 μm thick transverse and longitudinal sections, which were subsequently stained with a 0.5% w/v aqueous solution of safranin and 0.5% w/v aqueous solution of alcian blue. Sections were dehydrated in ethanol solutions of increasing concentration, and then mounted in Euparal (Carl Roth, Karlsruhe, Baden‐Württemberg, Germany). An exceptionally fragile sample of *D. della‐cellae* was fixed in 70% formalin–acetic acid–ethanol (2:1:7) solution, embedded in Technovit 7100 resin (Kulzer, Hanau, Hesse, Germany), and sectioned on a rotary microtome to obtain 7–10 μm thick transverse and longitudinal sections, which were stained with periodic acid–Schiff's (PAS) reagent without dinitrophenylhydrazine (DNPH). Wood anatomical characters were examined using light microscopy and follow the International Association of Wood Anatomists’ list of microscopic features for hardwood identification whenever possible (IAWA Committee, 1989). For traits that showed only limited variation, fewer character states were used.

A length‐on‐age curve was prepared only for *D. decipiens* because the samples of other species had wood cylinders that were too narrow. In *D. decipiens,* the wood was sliced into pieces 1 mm thick from the pith to the bark. Lengths of vessel elements and fibers were measured in each slice after maceration as described by Franklin (1945).

### Character evolution reconstruction

Reconstruction of the life history traits was conducted for all 60 species or subspecies included in the phylogenetic inference, while the reconstruction of wood anatomical traits was done for 21 species. All characters and their states are discussed in more detail in the Results section. Three discrete life history traits were assessed: (1) reproductive strategy (0, monocarpic; 1, polycarpic); (2) life span (0, annual or wintering annual; 1, biennial or triennial; 2, perennial); and (3) Raunkiær's life form (0, therophyte; 1, hemicryptophyte; 2, chamaephyte/rosette tree). Seven discrete wood anatomical traits were considered: (1) growth rings (0, absent; 1, only one ring; 2, distinct and recognizable); (2) wood porosity (0, semi‐ring‐porous; 1, diffuse‐porous); (3) ray formation (0, present in early wood; 1, delayed or rays absent); (4) rays composed mostly of upright and square cells (0, false; 1, true); (5) scalariform (and pseudoscalariform) intervessel pitting (0, uncommon; 1, common); (6) pervasive parenchyma (0, absent; 1, present); and (7) libriform fibers (0, absent; 1, present). We define “pervasive parenchyma” as abundant axial parenchyma replacing (at least partially) the imperforate tracheary elements in composition of the wood ground tissue (Carlquist, 2001, p. 172). We avoided the distinction between scalariform and pseudoscalariform intervessel pitting, because the only criterion proposed by Carlquist to distinguish these two types is not practically applicable in the studied group (“pseudoscalariform pitting looks like … the product of lateral elongation of pits in an alternate pattern”; Carlquist, 2001, p. 76).

As phylogenetic uncertainty was negligible, i.e., posterior probability for all essential internal nodes was high, we mapped life history and anatomical traits onto a MCC tree instead of taking into account multiple trees from the Bayesian analysis (e.g., Lens et al., 2016; Arévalo et al., 2017). For maximum parsimony reconstruction, we used the pace function from R package phangorn (Schliep, 2011) choosing unordered (Fitch) parsimony for all traits except for life span considered ordered character (Wagner parsimony). Maximum likelihood ancestral state estimates were calculated using the ace function in the R package ape (Paradis and Schliep, 2019) and the Akaike information criterion to select the best‐fitted model of evolution for each trait from three possible: equal rates (ER), symmetrical (SYM), all rates different (ARD). Similarly to the approach assumed in parsimony reconstruction, we applied a constraint of ordered evolution before selecting best‐fitted model for life span. This trait is markedly continuous, and it seems biologically unlikely that annual plants could evolve directly into long‐lived perennials without intermediate state of biennial form. Also the opposite—shortening of a life span from perennial directly into annual—seems biologically implausible and continuity of this trait has been demonstrated in natural populations (e.g., Hautekèete et al., 2002).

## RESULTS

### Phylogenetic relationships

In total, 35 new sequences were obtained for this study. The ETS marker was particularly variable providing over 20% of all parsimony informative sites, although its sequence was lacking for 77% of the species (Appendix S2). Sequences of molecular markers were partitioned into three groups: ITS, ETS, and chloroplast markers (*rpoB‐trnC* intergenic spacer and *rpoC1*,* rpl16*,* rps16* introns) with SYM + G, HKY + I, and GTR + I + G nucleotide substitution models, respectively. Although the relative likelihood ratio test returned *P* = 1.0 for the comparison of both nuclear markers—ITS and ETS—showing no evidence of incongruence in their phylogenetic signals, nuclear vs. plastid comparison rejected null hypothesis of congruence with *P* < 0.0001. However, excluding seven accessions identified by Banasiak et al. (2016) as responsible for topological differences at the generic level, mostly some species of *Thapsia* and *Laserpitium*, increased *p*‐value to 0.127, indicating that the topological conflict had a very limited scope.

The topology of the maximum clade credibility tree was generally congruent with earlier studies (Spalik and Downie, 2007; Banasiak et al., 2013, 2016), i.e., the previously designated sections within *Daucus*—*Daucus*,* Melanoselinum*,* Anisactis*, and *Agrocharis*—were retrieved. Posterior probabilities for most major clades were high (Fig. 3). The relationships among *Ekimia*,* Thapsia*,* Laserpitium* s.s., *Siler*, and *Laserocarpum* were somewhat different than in previous analyses of this group; however, these taxa are uniform with respect to their life form and life history. Hence, these differences did not affect subsequent analyses of these traits. *Daucus elegans* was resolved as sister to the clade of the Madeiran endemics (*D. decipiens* and *D. edulis*) with high support (posterior probability [PP] = 0.97). Previously placed in *Tornabenea*, the endemics of Cape Verde constituted a monophyletic group with *Daucus bischoffii* being sister to its former congeners: *D. annuus* and *D. tenuissimus* (PP = 1.0). These three species together with *D*. *insularis* formed a sister group to *D. carota*, while in previous studies, they were nested within the latter, albeit with a low support (Spalik and Downie, 2007; Banasiak et al., 2016). It is noteworthy that the predominantly monocarpic genera *Daucus*,* Silphiodaucus*, and *Orlaya* formed a crown clade, hereafter, named the mostly monocarpic clade (MMC), while the basal grade included perennial polycarpic species, hereafter, named the perennial polycarpic grade (PPG).

### Divergence time estimation and the age of insular lineages

Prior and posterior age distributions of the four nodes selected as calibration points differed to varying degree, although in all cases there was considerable overlap between the previous analyses and the current study (Fig. 3). The 95% confidence interval for the slope parameter of the major axis was 0.91–1.14. Therefore, there is no evidence for the systematic bias in secondary divergence time estimates as compared to primary calibration. The molecular markers differed substantially in the mean rate of substitution, with ETS having the fastest mean rate and plastid markers having the slowest rate (Table 2). Rate of molecular evolution also varied among branches, with about 2‐fold higher values for MMC than for PPG (Appendix S3).

**Table 2:** Clock rate statistics for ETS, ITS, and plastid markers used in phylogeny calibration.

Clock rate^a^	ETS	ITS	Plastid markers
Mean	5.12×10^–3^	4.35×10^–3^	8.35×10^–4^
SE	1.48×10^–5^	1.04×10^–5^	2.04×10^–6^
SD	5.53×10^–4^	3.46×10^–4^	6.43×10^–5^

a Number of expected substitutions per site per million years.

When estimating the age of insular endemics, we assumed that vicariance immediately followed dispersal—i.e., the time of dispersal from the continent to Macaronesia is equivalent to the age of the most recent common ancestor of the insular endemic clade and its continental sister group. The stem node age of the lineage encompassing the three Canarian‐Madeiran woody endemics (*D. elegans*,* D. decipiens*,* D. edulis*) was estimated at 8.55 million years (Myr) with 95% highest posterior density interval (HPD) of 9.85–7.44 Myr ago (Ma). The divergence between the Canarian *D. elegans* and the Madeiran endemics took place 7.77 Ma (95% HPD 9.05–6.35), while the age of the ancestor of Madeiran *D. decipiens* and *D. edulis* was estimated to be 3.54 Myr (95% HPD 5.32–1.73). The Canary Islands were likely colonized first, because they are closer to Africa than Madeira is; from there, the common ancestor of *D. decipiens* and *D. edulis* dispersed to the latter. The divergence between *D. carota* and the former genus *Tornabenea* occurred in the Pleistocene, 0.57 Ma (95% HPD 1.01– 0.25), and coincided with the dispersal from the continent to Cape Verde. The age of the most recent common ancestor of Cape Verde endemics was estimated to be 0.28 Myr (95% HPD 0.55–0.1), and *D. bischoffii* separated from its sister group 0.13 Ma (95% HPD 0.31–0.02). The ancestor of *D. carota* subsp. *azoricus* dispersed to the Azores 0.05 Ma with 95% HPD of 0.25–0.0, i.e., including the period of human colonization of the archipelago.

### General observations on wood anatomy

**Figure 4:**
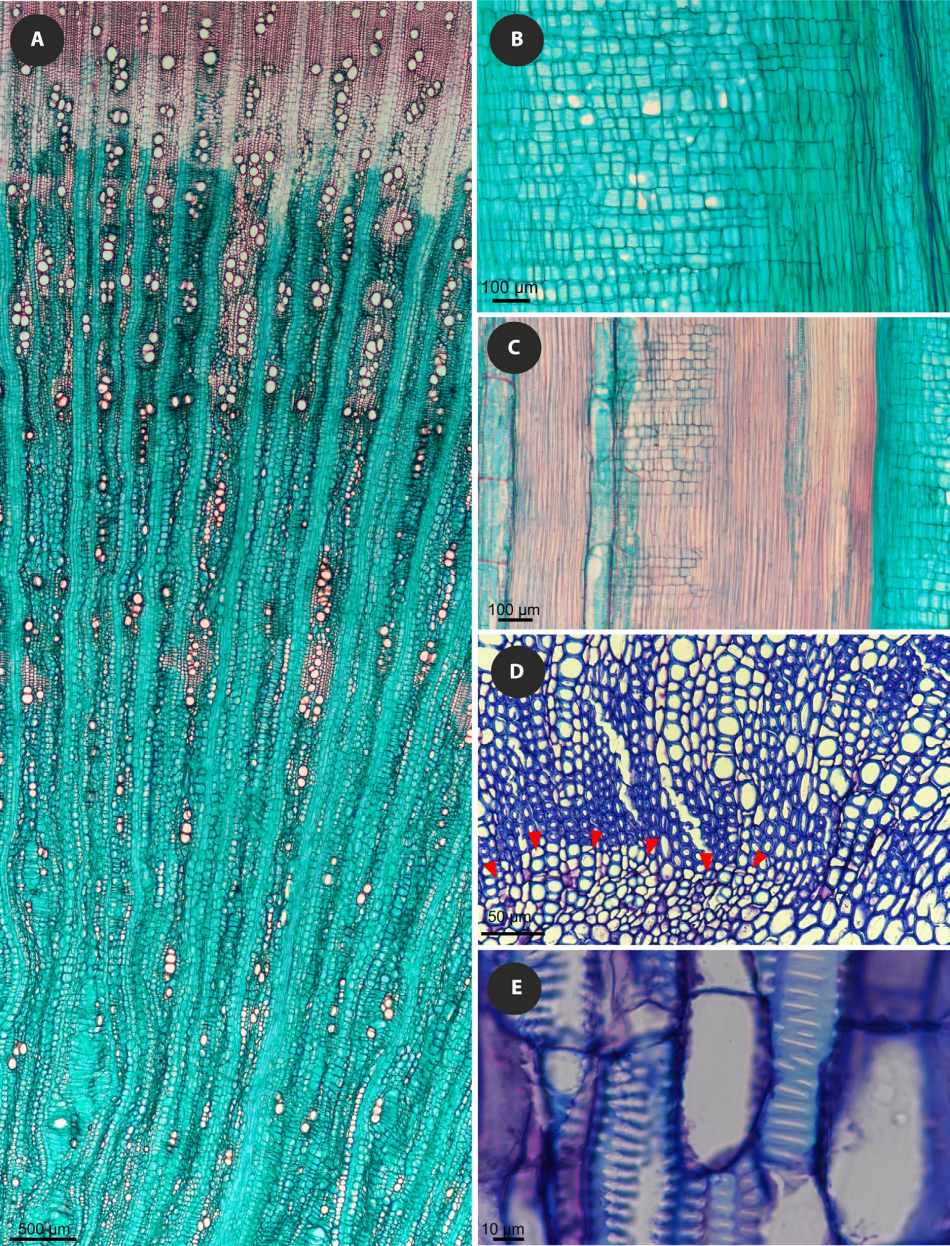
Wood anatomy of exemplar species with both parenchymatous and fibrous wood. (A–C) *Daucus edulis*. (D, E) *Daucus della‐cellae*. (A) Cross section showing transition from parenchymatous to fibrous wood. (B) Radial section illustrating mostly upright and square ray cells in parenchymatous wood. (C) Radial section showing more numerous procumbent ray cells in fibrous wood. (D) Cross section with visible transition from parenchymatous wood to wood of mixed constitution; the boundary is marked with red arrowheads. (E) Vessel elements with wide‐aperture scalariform intervessel pitting.

Wood anatomy allows for delineation of two groups: (1) those depositing parenchymatous wood or both parenchymatous and fibrous wood, (2) those depositing only fibrous wood. The first group comprises *D. della‐cellae*,* D. edulis*,* Laserpitium latifolium*, and *Silphiodaucus prutenicus* from among the newly examined species and most species, for which the data were retrieved from the xylem database (Schweingruber and Landolt, 2010). These species usually deposited wood cylinders composed of two distinct regions. Parenchymatous wood having abundant pervasive axial parenchyma without libriform fibers was located in the inner region of the secondary xylem cylinder, while fibrous wood with numerous libriform fibers was located in its outer region (Fig. 4). In most species, fibrous wood was predominant, whereas parenchymatous wood occurred only in the innermost part of the secondary xylem. In *D. edulis*, stem samples collected from vegetative shoots without floral buds had only parenchymatous wood, while samples from reproductive shoots had both parenchymatous and fibrous secondary xylem. *Daucus della‐cellae* had exclusively parenchymatous wood in the innermost region of the wood cylinder, whereas its outer wood had parenchymatous ground tissue with alternating bands of abundant and scanty fibers. Except for *D. carota* subsp. *gummifer*, all species for which data were obtained from the xylem database (Schweingruber and Landolt, 2010) fell into this category, having either exclusively parenchymatous wood (*Ferula communis*,* Laserpitium halleri*,* Siler montanum*,* Thapsia garganica*,* T. villosa*) or xylem with parenchymatous and fibrous regions (*Daucus pumilus*,* Laserpitium gallicum*,* L. peucedanoides*,* Orlaya grandiflora*).

**Figure 5:**
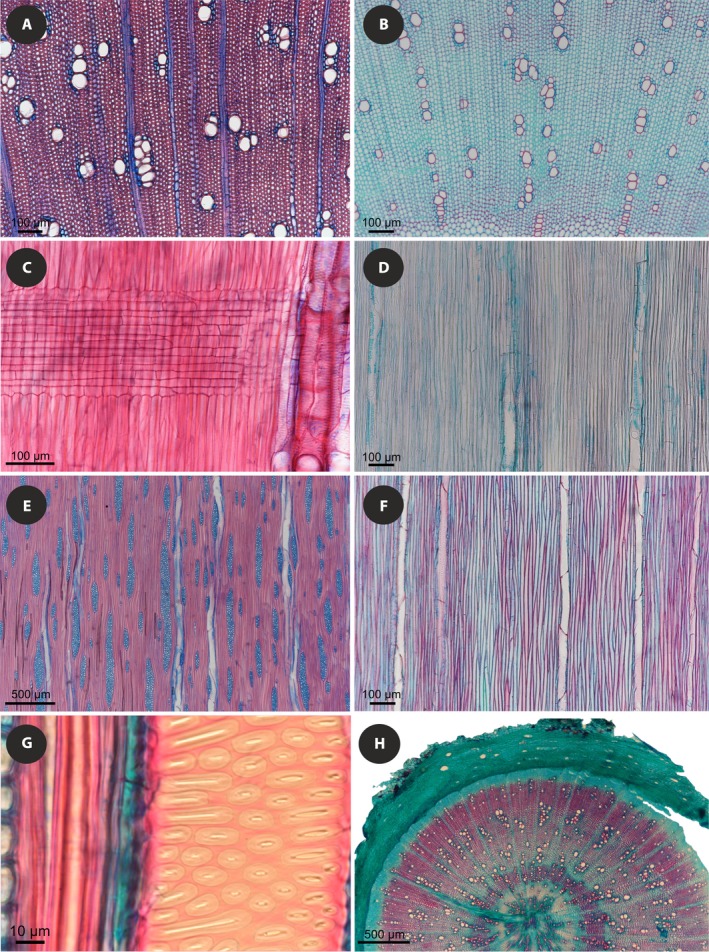
Wood anatomy of exemplar species with exclusively fibrous wood. (A, C, E, G) *Daucus decipiens*. (B, D, F) *Daucus elegans*. (H) *Daucus tenuissimus*. (A) Cross section showing prominent rays. (B) Rayless wood seen in cross section. (C) Radial section with numerous procumbent ray cells. (D) Rayless wood seen in radial section, simple perforation plates and axial parenchyma are clearly visible. (E, F) Tangential sections. (G) Mostly alternate to transitional intervessel pitting seen in tangential section. (H) Low magnification of the cross section with growth rings or density fluctuations.

The second group comprises *D. carota* (including *D. carota* subsp. *gummifer* from the xylem database), *D. decipiens*,* D. elegans*,* D. insularis*,* D. rouyi*,* D. tenuissimus*, and *D. bischoffii*. These species deposited exclusively fibrous wood cylinders (Fig. 5). It is noteworthy that both groups are polyphyletic and the insular treelets are in both: for example, Madeiran *D. edulis* produces both fibrous and parenchymatous wood, while its sister species, *D. decipiens*, has only fibrous wood.

Among the studied species, three paedomorphic traits were found: (1) delayed formation or absence of rays, (2) rays mostly composed of upright and square cells, and (3) scalariform intervessel pitting. The first character was observed only in *D. elegans*: our sample was completely rayless, while in the specimen studied by Schweingruber and Landolt (2010) the formation of rays was delayed. Rays mostly composed of upright and square cells usually occurred in species with a narrow (<1.5 mm) cylinder of secondary xylem, while *D. decipiens* and *D. edulis*, in which the wood cylinder was much wider (4–102 mm), had rays with more numerous procumbent cells. Scalariform intervessel pitting with wide pit apertures was found mostly in vessel element walls embedded in pervasive parenchyma constituting background tissue.

### Wood anatomy of newly studied species with both parenchymatous and fibrous wood

Growth rings are absent, i.e., wood is diffuse‐porous (Fig. 4). Vessels are mostly angular, occasionally rounded in outline (rounded vessels are more common in *D. edulis*). Mean vessel element length (not measured separately for parenchymatous and fibrous woods) ranges between 143 μm in *D. della‐cellae* and 274 μm in *D. edulis* (Appendix S4). Perforation plates are simple.

**Figure 6:**
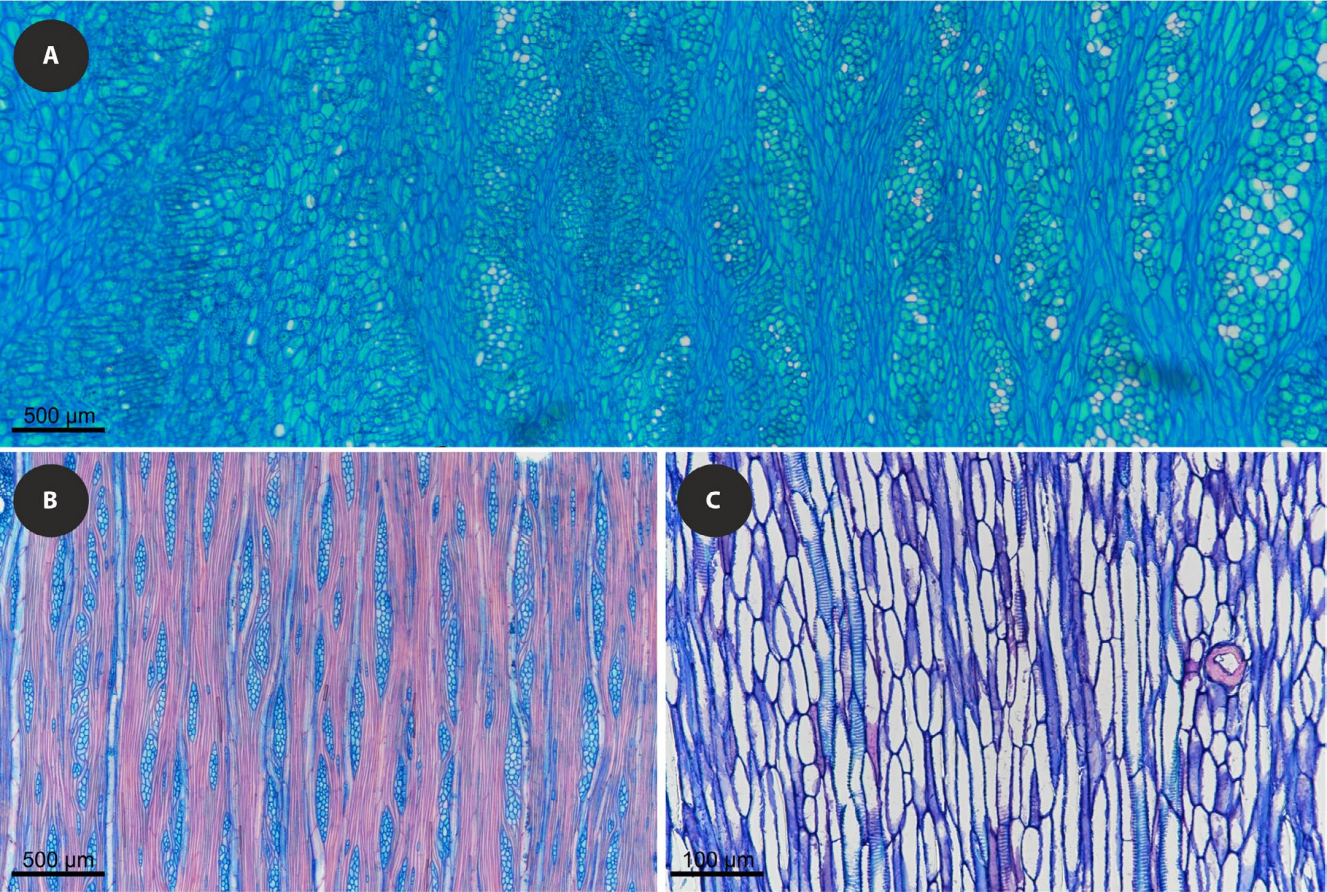
Tangential sections of (A) parenchymatous and (B) fibrous wood in *Daucus eduli*s, and (C) *Daucus della‐cellae*.

Rays are uniseriate and multiseriate, mostly 2–4 cells in width, up to 6–7 cells wide in *D. della‐cellae* and *D. edulis*, 9 cells wide in *L. latifolium* and 14 cells wide in *S. prutenicus*. The shortest multiseriate rays (<900 μm) were found in *D. della‐cellae* and the tallest ones (>4000 μm) in fibrous wood of *D. edulis*. They are composed of exclusively upright and square cells in *D. della‐cellae* and mostly upright cells with square and procumbent cells mixed throughout the rays in *L. latifolium*,* S. prutenicus*, and parenchymatous wood of *D. edulis*. In fibrous wood of *D. edulis*, multiseriate rays consist mostly of procumbent cells, with square and procumbent cells in 1–2 marginal rows and solitary rows alternating with procumbent cells or as solitary sheath cells (Fig. 6). Scarce radial secretory canals occur only in *D. edulis* and *S. prutenicus*. Calcium oxalate crystals were not found in ray cells.

#### Parenchymatous wood

Vessels vary from very narrow in *D. della‐cellae* (tangential diameter 5–19 μm), to narrow in *D. edulis* (25–82 μm) and *L. latifolium* and *S. prutenicus* (<55 μm). Vessels are few in *D. edulis* (16 mm^–^²), more numerous in *S. prutenicus* (64 mm^–^²) and *L. latifolium* (124 mm^–^²), and very numerous in *D. della‐cellae* (206 mm^–^²), mostly arranged in small clusters and radial to diagonal multiples (2–5 vessels) in *D. edulis* and in larger groupings in other species (up to 18 vessels in *L. latifolium*). Vessel walls are thin in *D. della‐cellae* (1.0–3.8 μm thick), thicker (>2.2 μm thick) in *L. latifolium* and *S. prutenicus*, and thickest in *D. edulis* (up to 9.4 μm). Intervessel pitting is exclusively scalariform in *D. della‐cellae*, and mostly scalariform, occasionally transitional to alternate, and alternate in other species. Opposite pitting occurs in *S. prutenicus*. Intervessel pits are minute to small (1.8–6.8 μm in vertical size), mostly rounded (occasionally polygonal in *D. edulis*), and with wide lens‐like apertures. Vessel‐ray and vessel‐axial parenchyma pits are distinctly bordered and usually similar in size and shape to intervessel pits (mostly opposite in *L. latifolium*). Helical thickenings were not found, nor were vascular tracheids. Axial parenchyma is pervasive and scanty paratracheal in strands of 2–4 (*D. della‐cellae*,* S. prutenicus*) or 3–5 cells (*D. edulis, L. latifolium*).

#### Fibrous wood

Vessels are very narrow in *D. della‐cellae* (7–34 μm) and *L. latifolium* (10–51 μm) and narrow in *D. edulis* (20–70 μm) and *S. prutenicus* (8–61 μm), few in *D. edulis* (22 mm^–^²) and *L. latifolium* (34 mm^–^²), more numerous in *S. prutenicus* (86 mm^–^²), and very numerous in *D. della‐cellae* (477 mm^–^²). Vessels are mostly arranged in clusters and radial to diagonal multiples (2–8 vessels, up to 12 vessels in *D. edulis*). Vessel walls are thin in *D. della‐cellae* and *S. prutenicus* (1.1–3.3 μm and 1.3–4.7 μm thick, respectively) and thicker (mostly 1.8–6.5 μm) in other species. Intervessel pitting is almost exclusively scalariform (occasionally alternate) in *D. della‐cellae*, and mostly alternate, occasionally scalariform or transitional to alternate, in other species. Opposite pitting occurs in *S. prutenicus*. Intervessel pits are minute to small (1.8–7.8 μm in vertical size), rounded, and usually have narrow slit‐like apertures (in *L. latifolium* lens‐like). Vessel‐ray and vessel‐axial parenchyma pits are distinctly bordered, similar in size and shape to intervessel pits. Helical thickenings were not found, nor were vascular tracheids.

Fibers are libriform, thin‐ to thick‐walled (0.9–2.5 μm thick in *D. edulis* and between 1.1–4.1 μm thick in other species), nonseptate, and with simple to minutely bordered pits located in radial and tangential walls. Mean fiber length ranges from 207 μm (in *D. della‐cellae*) to 415 μm (in *S. prutenicus*). Axial parenchyma is scanty paratracheal (in *L. latifolium* also diffuse) in strands of 2–4 (*D. della‐cellae*,* S. prutenicus*) to 3–5 cells (*D. edulis*,* L. latifolium*).

### Wood anatomy of newly studied species with exclusively fibrous wood

Growth rings are generally indistinct, except for some samples of *D. decipiens*,* D. bischoffii*, and some sections of *D. tenuissimus* that are marked by differences in lignification of fiber walls between early‐ and latewood detected by safranin staining (Fig. 5). Wood is diffuse‐porous. Vessels are very narrow (<45 μm) in all species, except for *D. insularis*, where they are narrow (≤70 μm); they are few in *D. rouyi* and *D. insularis* (<50 mm^–^²), more numerous in *D. decipiens* and *D. elegans* (ca. 70 mm^–^²), and very numerous in *D. bischoffii* (159 mm^–^²), *D. carota* (176 mm^–^²) and *D. tenuissimus* (214 mm^−2^). Vessels are arranged mostly in small clusters and radial or diagonal multiples, which vary in number of vessels: 3–5 in *D. elegans*, up to 8 in *D. carota*, up to ca. 12 in *D. decipiens, D. insularis*,* D. rouyi*,* D. bischoffii*, and up to 23 in *D. tenuissimus*. Solitary vessels are mostly angular (*D. decipiens*,* D. elegans*,* D. tenuissimus*), angular to rounded (*D. insularis*,* D. bischoffii*), or mostly rounded (*D. rouyi*). Vessel walls are 2.3–6.8 μm thick in *D. rouyi*, and thinner (≤4.6 μm thick) in other species. Mean vessel element length ranges from 216 μm (*D. rouyi*) to 481 μm (*D. decipiens*). Perforation plates are simple. Intervessel pitting is mostly opposite (occasionally alternate and scalariform) in *D. rouyi*, alternate (occasionally transitional to scalariform) in *D. bischoffii*, alternate to scalariform in *D. tenuissimus*, and mostly alternate (occasionally opposite and transitional to scalariform) in other species. Intervessel pits are minute to small (3.1–7.0 μm in vertical diameter), rounded, with narrow slit‐like apertures. Vessel‐ray and vessel‐parenchyma pitting is mostly similar to intervessel pitting in size and shape, distinctly bordered, and mostly alternate; in *D. decipiens* and *D. rouyi*, it is sometimes scalariform. Helical thickenings were not found, nor were vascular tracheids.

Fibers are libriform, thin‐ to thick‐walled, mostly between 1–3 μm thick, up to 2.3 μm thick in *D. elegans,* up to 3.7 μm thick in *D. tenuissimus*, and up to 4.3 μm thick in *D. bischoffii*, mostly nonseptate (septate fibers occur in *D. bischoffii*) with simple to minutely bordered pits in radial and tangential walls. Mean fiber length ranges from 247 μm (*D. rouyi*) to 530 μm (*D. decipiens*).

Axial parenchyma is scanty paratracheal, in strands of 2–9 (*D. decipiens*), 3–6 (*D. rouyi*,* D. tenuissimus*,* D. bischoffii*), 8–10 (*D. carota*) or 8–15 cells (*D. elegans*,* D. insularis*).

Rays are absent in *D. elegans*, while in other species they are uniseriate and multiseriate with an average of 4 cells in width, up to 5 in *D. carota*,* D. insularis*, and *D. tenuissimus*, up to 9 in *D. rouyi* and *D. bischoffii*, and up to 15 in *D. decipiens*. In *D. carota* and *D. rouyi*, multiseriate ray height exceeds the length of the entire section (>2 mm and >4 mm, respectively). In the remaining species, mean height ranges between 342 μm (*D. decipiens*) and 689 μm (*D. bischoffii*). Rays are composed of procumbent cells, sometimes with square and upright cells in 1–3 marginal rows in *D. decipiens*, or exclusively of upright and square cells in *D. carota*,* D. insularis*,* D. rouyi*,* D. tenuissimus*, and *D. bischoffii*. Uniseriate rays are made of procumbent cells or also upright and square cells in *D. decipiens* and of square and upright cells in other species. Few radial secretory canals were found only in *D. bischoffii*. The length‐on‐age curve for *D. decipiens* is ascending (Appendix S5).

### Phylogenetic character mapping

**Figure 7:**
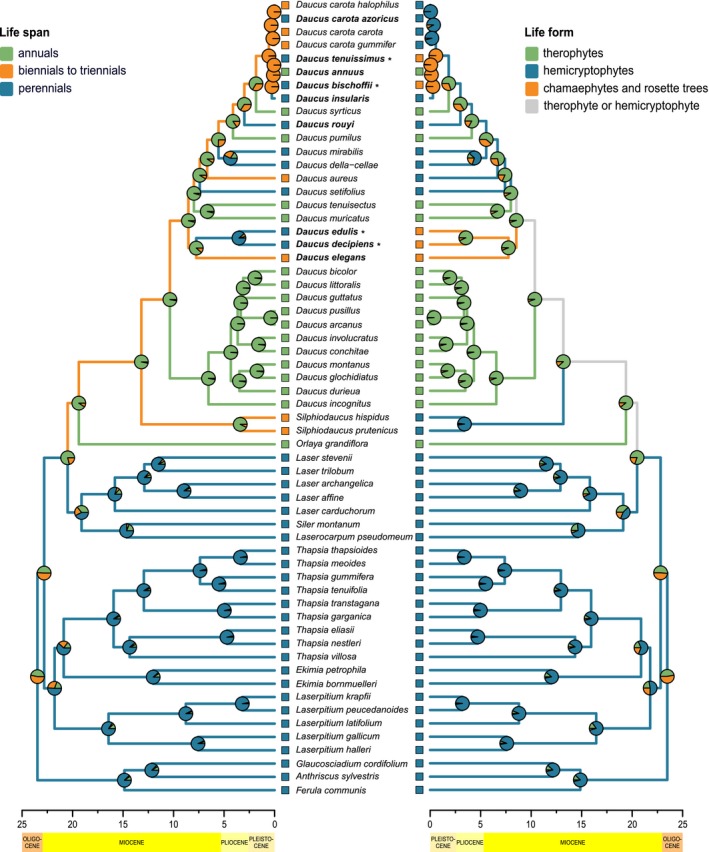
Life form and life span evolution in Daucinae reconstructed using maximum parsimony (branch colors) and maximum likelihood estimates of ancestral states (pie charts represent relative likelihoods). Time scales are given in millions of years ago. Insular species are marked in boldface; insular rosette treelets are marked with asterisks. The model all rates different (ARD) was chosen as the best fit for both traits, and the constraint of ordered evolution was applied for the life span character.

**Figure 8:**
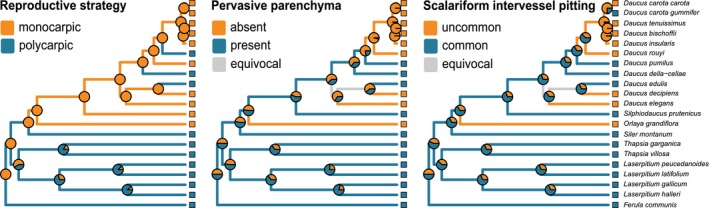
Evolution of reproductive strategy, the presence of pervasive parenchyma, and the occurrence of scalariform intervessel pitting reconstructed using maximum parsimony (branch colors) and maximum likelihood estimates of ancestral states (pie charts represent relative likelihoods). The model equal rates/symmetrical (ER/SYM) was chosen as the best fit for evolution of reproductive strategy; the all rates different (ARD) model was chosen for all remaining traits.

The results of ancestral state reconstruction differed substantially between the two methods used. Parsimony resolved the most recent common ancestor of Daucinae as polycarpic perennial hemicryptophyte (Figs. 3, 7, and 8; Appendix S6), and these plesiomorphic character states were retained in the basal grade comprising *Laserpitium*,* Ekimia*,* Thapsia*,* Siler*, and *Laser* (PPG). A clade of *Orlaya*,* Silphiodaucus*, and *Daucus* is predominantly monocarpic (MMC) with four reversals to polycarpy inferred for *D. edulis*,* D. setifolius*,* D. rouyi*, and a clade formed by *D. della‐cellae* and *D. mirabilis* (Fig. 3; Appendix S6).

Parsimony reconstructed the most recent common ancestor of genus *Daucus* as a monocarpic biennial to triennial therophyte or hemicryptophyte. However, because therophytes are annual by definition, one may exclude the combination of biennial/triennial life span and therophyte life form. The same character states were inferred as ancestral for the most recent common ancestor of sect. *Daucus* and *Melanoselinum* with highly homoplastic subsequent evolution of six episodes of life span prolongation (from biennial or triennial to perennial) and four independent cases of life span shortening (three of them from biennial or triennial to annual and one from perennial to annual; Fig. 7).

Parsimony revealed two independent shifts to the chamaephyte/rosette tree life form, which occurred in the Canarian‐Madeiran lineage (*D. elegans*,* D. decipiens*,* D. edulis*) and in the species of *Daucus* from Cape Verde, formerly classified in *Tornabenea* (Fig. 7).

On the other hand, the maximum likelihood estimate for the most recent common ancestor of Daucinae was a monocarpic annual therophyte or biennial/triennial chamaephyte, while the most recent common ancestor of genus *Daucus* was inferred as a monocarpic annual therophyte (Figs. 3 and 7).

In *Daucus*, delayed formation of rays is autapomorphic for *D. elegans*, while the presence of mostly upright and square ray cells is synapomorphic for the Madeiran clade (not shown). Regardless of the ancestral state estimation method, the most recent common ancestor of *Daucus* was reconstructed as lacking growth rings, diffuse porous species with libriform fibers. (This is further supported by presence of such fibers in all studied *Daucus* species, although not necessarily throughout the whole life span; Appendix S6.)

Presence of pervasive parenchyma and scalariform intervessel pitting was reconstructed with MP as plesiomorphic for *Daucus*, while ML method did not exclude any scenario, i.e., relative likelihoods of alternative character states were comparable. Pervasive parenchyma co‐occurred with scalariform intervessel pitting except for *Daucus carota* subsp. *gummifer* , and both traits were more common in polycarpic species than in monocarpic ones (Fig. 8).

## DISCUSSION

### Dispersal of *Daucus* to Macaronesia

Including the ETS marker in the phylogenetic analyses helped resolve the positions of two chamaephytic species, *D. elegans* and *D. bischoffii*, that are crucial for understanding life‐form evolution and dispersal patterns in Daucinae. The first one was sister to the Madeiran endemics, whereas the latter was placed together with other species formerly recognized in *Tornabenea*. The results of our study (Fig. 3) corroborate the biogeographic scenario inferred by Spalik and Downie (2007), which postulated three independent dispersals of *Daucus* to Macaronesia that gave rise to (1) the Canarian‐Madeiran clade encompassing *D. elegans*,* D. decipiens*, and *D. edulis*; (2) the Cape Verde clade including species formerly recognized in *Tornabenea*; and (3) the Azorean *D. carota* subsp. *azoricus*. Because the Canary Islands are closer to Africa than Madeira, the former archipelago was probably colonized first, and from there, the common ancestor of *D. edulis* and *D. decipiens* dispersed to Madeira. Such a dispersal pattern is contrary to the dominant wind pattern, but has been uncovered for many Macaronesian endemic clades (e.g., *Crambe*,* Convolvulus*,* Sonchus*; Francisco‐Ortega et al., 2002; Carine et al., 2004; Lee et al., 2005). A second colonization of Macaronesia—from the continent to Cape Verde—led to the radiation of the former genus *Tornabenea*. A third and a very recent dispersal gave rise to *D. carota* subsp. *azoricus*. Given its young age with 95% HPD encompassing modern times and the results of genomic studies that do not support *D. carota* subsp. *azoricus* as a separate taxon (Arbizu et al., 2016), it is probable that this taxon colonized the Azores with humans.

### Incongruence between parsimony and ML reconstructions

The most important difference between MP and ML methods is that the latter accommodate for branch lengths, while parsimony neglects this information. Hence, ML infers changes as more probable on long branches rather than on short ones. Because branch lengths of a phylogenetic tree may be scaled to time (chronograms) or to the expected number of nucleotide substitution (phylogram), there comes a question of which one to use, or whether to abandon this information altogether (Pagel, 1999). Unlike the case for the molecular data, there is no good evidence that morphological traits, which often are subject to natural selection, evolve stochastically as is assumed by Markov models employed by ML (Tuffley and Steel, 1997; Cunningham et al., 1998; Goloboff et al., 2019). Moreover, selection of chronogram or phylogram for ML reconstruction may provide very distinct results, as is actually the case in the present study, where ML reconstruction with chronogram inferred ancestral states different from the ones resolved by MP, while the estimation using a phylogram (not shown) was in general congruent with MP. On the other hand, parsimony tends to be misleading when rates of evolution are high and when the probabilities of gains and losses are not equal (Cunningham et al., 1998). For these reasons, the results of ancestral state reconstruction should be interpreted with caution.

### Habit evolution and the lability of wood ground tissue

Parsimony‐reconstructed ancestral combination of traits for the most recent common ancestor of genus *Daucus* (as well as for the ancestor of sections *Daucus* and *Melanoselinum*) was biennial to triennial, monocarpic therophyte or hemicryptophyte. Since therophytes are annuals, it is reasonable to assume that the hemicryptophytic habit is plesiomorphic for the genus and its typical section (i.e., *Daucus* sect. *Daucus*). Life span is an ecologically labile trait: in certain conditions, the usually biennial carrot (*Daucus carota*) may flower and die in its first year or it may postpone blooming until the third year (Lacey, 1988). The combination of character states reconstructed for *Daucus* is intermediate between annual therophytes and perennial hemicryptophytes or chamaephytes/rosette treelets, thereby enabling subsequent evolution in both directions and for this reason is congruent with the observed pattern of life span and life form in the genus.

The chamaephyte/rosette treelet life form arose at least twice: in the most recent common ancestor of *Daucus elegans* and *Daucus* sect. *Melanoselinum* (*D. decipiens* and *D. edulis*) and in the former genus *Tornabenea* within sect. *Daucus*. Such repetitive evolution has been documented multiple times on islands, including at least 38 cases on the Canary Islands alone (e.g., Böhle et al., 1996; Kim et al., 2008; Lens et al., 2013b; Nürk et al., 2019). Aside from Daucinae, other cases of insular woody apioids include *Nirarathamnos asarifolius* Balf. f. from Socotra and *Angelica lignescens* Reduron & Danton from the Azores (Press and Dias, 1998; Oskolski, 2001).

Two sister species comprising *Daucus* sect. *Melanoselinum* represent strikingly different stem anatomies (solely fibrous wood in *D. decipiens* vs. parenchymatous and fibrous wood in *D. edulis*; Figs. 4 and 5) and life histories (monocarpic vs. polycarpic; Fig. 8). Hence, it cannot be precluded that chamaephytic/rosette treelet habit evolved in this clade twice or even three times (when the closely related *D. elegans* is also taken into consideration). This scenario is corroborated by the case of very rapid evolution of the chamaephyte with fibrous wood in stem, *D. bischoffii*, which originated ca. 130,000 years ago—suggesting that habit shifts within *Daucus* can occur rapidly—and by the ML reconstruction of ancestral life form. The stem node age of *Daucus* sect. *Melanoselinum* is 8.5 Myr (95% HPD 9.85–7.44), which would have allowed many evolutionary changes to have taken place. As demonstrated for *Arabidopsis thaliana*, the formation of secondary xylem cylinder may occur through mutations in only two genes regulating the timing of flowering (Lens et al., 2012b), which points to the lability of this trait. However, only through genomic studies can we possibly answer the question of whether *D. decipiens* and *D. edulis* evolved the chamaephytic habit independently or inherited it from their most recent common ancestor.

Among the chamaephytic species, *D. decipiens*,* D. elegans*,* D. bischoffii*, and *D. tenuissimus* have fibrous wood, whereas *D. edulis* has abundant pervasive parenchyma in the inner region of the wood cylinder (Figs. 4 and 5). Libriform fibers are present also in secondary xylem of all newly studied species, but the amount varies considerably from an entire cylinder of fibrous wood to narrow bands or clusters of fibers embedded in pervasive parenchyma. Our data show, therefore, that the shifts to chamaephytes are not necessarily associated with an increase in fibrous wood deposition. Generally, all wood anatomical traits of insular woody species of *Daucus* can be found also in other species of this genus, e.g., their ground tissue may be composed either of fibers alone or of pervasive parenchyma and fibers together. In other plant groups, the evolutionary shift between herbaceous and woody life forms is also not strictly linked to the evolution of specific wood traits (Stepanova et al., 2007; Schweingruber and Büntgen, 2013) and similar diversity of wood traits among related species with unlike habits is also found in *Bupleurum*, another genus of umbellifers (Stepanova and Oskolski, 2010).

### Causes of insular woodiness in *Daucus*

The shifts to chamaephytic life form in the clade comprising Canarian *D. elegans* and Madeiran *D. decipiens* and *D. edulis* and in the former genus *Tornabenea* from Cape Verde represent cases of insular woodiness. To explain such habit shifts, Lens et al. (2013b) hypothesized that derived woodiness evolved as a result of selection for greater air embolism resistance in the vascular system. This hypothesis has recently been supported by ecophysiological data on shrubby species of *Argyranthemum* from the dry lowlands of the Canary Islands (Dória et al., 2018). This explanation, however, is not plausible for the chamaephytic Daucinae, as none of the insular endemic species of this tribe is confined to dry coastal regions of the Macaronesian islands. Instead, these species occur in more humid and mild habitats, usually at higher elevations (Martins, 1996; Brochmann et al., 1997; Press and Dias, 1998; Fernandes and Carvalho, 2014; GBIF.org, 2018). The evolution of derived woodiness in *Daucus* may also be explained by other factors of insular environment promoting longer life span, such as competition for pollinators in a pollinator‐poor setting and release from large herbivore pressure (reviewed by Carlquist, 1972; Jorgensen and Olesen, 2001; Dulin and Kirchoff, 2010; Whittaker et al., 2017; see also Darwin, 1859; Wallace, 1878) or the effect of moderate climate (Carlquist, 1974). Assuming that biennial/triennial (MP reconstruction) or annual (ML reconstruction) habit was plesiomorphic for the whole genus, the prolonged life span hypothesis may account for the treelet life form of Madeiran and Cape Verde perennials. In case of *D. elegans* from the Canary Islands where prominent dry season occurs (Mies, 1995; Cropper, 2013), embolism resistance is also a plausible explanation for evolution of woodiness, especially that this species has not evolved markedly longer life span.

### Reproductive strategy and wood anatomical traits

Reproductive strategy, pervasive parenchyma, and scalariform intervessel pitting seem to covary when reconstructed on a phylogenetic tree (Fig. 8). This relationship was asserted in previous studies (e.g., Sibout et al., 2008; Ragni et al., 2011). We additionally calculated Pagel's correlations (1994; as implemented in Mesquite) between each pair of these traits, i.e., reproductive strategy, presence of pervasive parenchyma, and presence of scalariform intervessel pitting, which were statistically significant (*P* < 0.01). However, Maddison and FitzJohn (2015) demonstrated that current tests for phylogenetic correlation between categorical characters—including Pagel's (1994) test—do not eliminate pseudoreplication because they are susceptible to an effect of a single coevolutionary event inherited by multiple descendants.

Daniel (1916) and Radkevich (1928) suggested that shortening of the internodes and formation of a leaf rosette during the vegetative phase of shoot growth might be responsible for development of parenchymatous zones in the secondary xylem, while the transition to flowering triggers a shift to the formation of wood. Sibout et al. (2008) showed that flowering might be responsible for xylem expansion in hypocotyl and root of *Arabidopsis*. Ragni et al. (2011) broadened this view by presenting evidence that, in plants with leaf rosettes, the appearance of fibers never occurred before flowering, unlike in related species without rosettes. The authors studied selected species of Asteraceae and Brassicaceae, but leaf rosettes are a common feature of Daucinae (Table 1), making a similar scenario plausible also in this group. The mechanism of interdependence between flowering and fibers deposition remains obscure, but it is clear that this connection exists (Chaffey et al., 2002; Lens et al., 2012b). The formation of fibrous wood may be induced by gibberellic acid, which plays an important role both in the developmental switch to flowering (Conti, 2017) and in stimulating secondary xylem differentiation and expansion of cambial derivatives, including fiber elongation (Moritz et al., 2000; Israelsson et al., 2005; Mauriat and Moritz, 2009; Ragni et al., 2011; Strabala and MacMillan, 2013; Ye and Zhong, 2015; Ragni and Greb, 2018). If so, the shift from parenchymatous to fibrous wood could be explained by the switch from vegetative to reproductive growth in the plant life history.

Among species for which wood anatomical data were available in the study, parenchymatous wood was often concomitant with polycarpic reproductive strategy, while monocarpic species deposited solely fibrous wood. This pattern could be explained by longer vegetative growth in the former group, followed by floral shift leading to deposition of libriform fibers. In the monocarps, flowering shift occurs early in plant development leading to the deposition of fibrous wood from the beginning of secondary growth. Such a shift may also have mechanical explanation because fibrous wood provides more support for orthotropic shoots with long internodes and terminating in heavy inflorescences (Ko et al., 2004; Sibout et al., 2008).

Simultaneously, the co‐presence of pervasive parenchyma and scalariform intervessel pitting has been observed in other groups, e.g., *Phacelia* (Boraginaceae; Carlquist and Eckhart, 1984), *Potentilla* (Rosaceae; Stepanova et al., 2007), *Bupleurum* (Apiaceae; Stepanova and Oskolski, 2010), and also in succulents including Crassulaceae (Carlquist, 2001, 2009b) and Cactaceae (Gibson, 1973). The shift to wide scalariform intervessel pit apertures might be the result of relaxation of mechanical constraints in stems supported primarily by turgor of parenchyma cells, which may be an adaptation to expansion and contraction of the stem with fluctuating turgor (Carlquist, 2009b; Lens et al., 2012a; Kedrov, 2013).

## CONCLUSIONS

The most likely ancestral habit of *Daucus* was biennial to triennial monocarpic hemicryptophyte, and these traits were retained in the most recent common ancestor of sect. *Daucus* and *Melanoselinum*. This habit makes a convenient starting point for both shortening and extending of the life span and is congruent with observed highly homoplastic evolution of habit. At least two independent shifts to chamaephytes/rosette treelets occurred in the genus and represent cases of insular woodiness in Canarian‐Madeiran lineage of *D. elegans*,* D. decipiens*, and *D. edulis*, and, a very rapid one, in former *Tornabenea* of Cape Verde. Wood anatomy of insular, “woody” species show the same set of traits as observed among their continental, “herbaceous” relatives suggesting that in *Daucus*, life‐form evolution is not constrained by any features of their wood structure. At the same time, sister species of rosette treelets—*D. decipiens* and *D. edulis*—have strikingly different stem anatomies. This dissimilarity opens a possibility of treelet life form evolving multiple times in the Canarian‐Madeiran lineage. For these reasons, *Daucus* makes an interesting model for further testing of hypotheses regarding evolution of insular woodiness.

We observed that monocarpic species tend to have only fibrous wood, while polycarpic species deposit parenchymatous and fibrous xylem, and that scalariform intervessel pitting is predominantly present in parenchymatous wood. Although it is possible that a common mechanism, similar to that advocated in previous studies, is responsible for development of these three traits, the limited number of sampled species preclude making any definitive statements.

## AUTHOR CONTRIBUTIONS

K.F. prepared and analyzed wood samples, gathered the ecological and morphological data, performed phylogenetic analyses, and wrote the manuscript. A.O. supervised wood anatomical analyses and wrote the manuscript. Ł.B. supervised the phylogenetic analyses and reviewed the manuscript. F.F., J.‐P.R., JARB collected samples in the field and provided information about their ecology. L.S., M.A., and J.B. obtained new DNA sequences. K.S. designed and supervised the study, and edited the manuscript. The authors have no conflicts of interest to declare.

## Supporting information


**APPENDIX S1.** Accession table.

**APPENDIX S2.** Characteristics of the data set used in phylogenetic analysis table.

**APPENDIX S3.** ITS and plastid markers median clock rate values.

**APPENDIX S4.** Wood trait measurements.

**APPENDIX S5.** *Daucus decipiens* length‐on‐age curve.

**APPENDIX S6.** Results of ancestral state estimation for growth rings, wood porosity, libriform fibers, and reproductive strategy.

## Data Availability

Matrix with aligned molecular sequences before and after trimming, maximum clade credibility tree, and data matrices with habit and anatomical data are available online in TreeBase (http://purl.org/phylo/treebase/phylows/study/TB2:S24902?x-access-code=c8c88a0b3b02f32f309a4bb8221d0568%26format=html).
