# Supplementary material for: Parallel evolution of arborescent carrots (Daucus) in Macaronesia
Source: Am J Bot. 2020 Mar 8;107(3):394–412. doi: 10.1002/ajb2.1444 (PMC7155066; doi:10.1002/ajb2.1444)
Supplement: Supplementary file 1 — APPENDIX S1. Accession table. [file AJB2-107-394-s001.pdf]

Appendix S1 Accessions of Scandiceae subtribe Daucinae and three species used as outgroups with corresponding accession identifiers, voucher and/or reference information, and GenBank reference numbers. All herbarium acronyms are according to Index Herbariorum (Thiers, 2013). Letter "A" following accession identifier indicates specimen used for anatomical study and "v" and "g" mark vegetative or generative stage, respectively, of sampled plant. Newly obtained sequences are marked with asterisks preceding GenBank accession numbers.

**Taxon**	**Accession ID**	**Voucher information**	**GenBank accession numbers**					
			ETS	ITS	*rpoB-trnC* spacer	*rpoC1* intron	*rpl16* intron	*rps16* intron
**Outgroup**								
Anthriscus sylvestris (L.) Hoffm.	0083	France, Haut-Rhin, Mulhouse, Forêt du Tannenwald, 8-Sep-1973, *Reduron* no. 19730908-01 (Reduron, pers. coll.)		KT347715	KT347743	KT347806		KT347879
	G135	ZJ0566 (KUN)					FJ385078	
Ferula communis L.	0195	Spain, Almería, Vélez-Rubio, 12 Jun 2004, *Sánchez-Gómez s.n.* (Univ. of Zaragoza, Spain)		DQ379392	KJ660616	KJ660477		KJ698369
Glaucosciadium cordifolium (Boiss.) B.L.Burtt & P.H.Davis	0221	Cyprus, Paphos district, cult. Cons. bot. Mulhouse no. 98112, 8 Dec 1999, *Reduron s.n.* (WA 0000050884)		DQ379459	KJ660745	KJ660458		KJ660439
**Ingroup**								
Daucus annuus (Bég.) Wojew., Reduron, Banasiak & Spalik	0521	Cape Verde Islands, cult. Cons. bot. Mulhouse no. 99145, 9 Sep 2000	*MK142912	KT347712	KT347742	KT347803		KT347877
	2209	Cape Verde Islands, cult. Cons. Bot. Mulhouse no. 98115					*MK142855	
Daucus arcanus García-Martín & Silvestre	0260	Mexico, Baja California, Colonia Vincente Guerrero, 20 m, 28 Apr 1980, *Davis & Lightowlers* 66231 (E) [specimen labelled as *D. pusillus*]		KT347671				KT347858
Daucus aureus Desf.	G018	Spain, Córdoba, Arroyo La Maturra, Baena, 250 m, 12 Jun 2009, *Triano & Castro s.n.* (ABH 55117)		JQ290119				JQ290131

Taxon	Accession ID	Voucher information	GenBank accession numbers ETS	ITS	*rpoB-trnC* spacer	*rpoC1* intron	*rpl16* intron	*rps16* intron
Daucus bicolor Sibth. & Sm.	0041	Israel, Judean Mountains, Har Herzel, near Jerusalem, shrubs formation, terra rosa soil, 800 m, 2 Aug 1986, *Cohen s.n.* (WA 0000050890)		KT347652		KT347777		KT347843
Daucus bischoffii (J.A.Schmidt) Spalik & Banasiak	2060 Ag	Cape Verde Islands, S.Antao, Rib. dos Orgaos, West side of Riberias, 450 m, 9-Dec-1985, *N.Kilian 799* (FR 0029088) [specimen labelled as *T. bischoffii*]	*MK142899	*MK142913			*MK142842	*MK142885
	2061 Ag	Cape Verde Islands, Santiago Island, Santa Catarina district, Boa Entrada, 1980, *J. Spatz s.n.* (FR 0029089) [specimen labelled as *T. bischoffii*]						
Daucus carota subsp. *azoricus* Franco	0610	Portugal. Azores, Ilha do Pico, Porto do Calhau, 21 Jul 2009, *Danton s.n.* (Cons. bot. Mulhouse no. 08-092A; Reduron, pers. coll.)		KT347693				KT347869
Daucus carota L. subsp. *carota*	0032	France, Corsica, cracks of the rocks of Porto, 20 Jul 2009, *Reduron s.n.* (Cons. bot. Mulhouse no. 08-114A; Reduron, pers. coll.; WA 0000050896)		KT347694		KT347798		KT347870
	2057 Ag	Poland, Łódź Voivodeship, Łowicz County, Łowicz Commune, 40 m SE from railway crossing of the road leading from Niedźwiada village to the trunk road no. 92 (former 2) and a railway line from Łowicz to Kutno, at the roadside 52.135157 N 19.901009 E, *Ł. Banasiak s.n.* (WA 0000050198)						
Daucus carota subsp. *gummifer* Hook.f.	0445	France, Pyrenees, Banyuls-sur-Mer, 27 Jun 1962, *Kohlmeyer 1448* (B)		KT347699				KT347874
Daucus carota subsp. *halophilus* (Brot.) Okeke	0031	Portugal, Cabo Sardão, cult. Cons. bot. Mulhouse no. 9305, 17 Jun 1993		KT347700		KT347801		KT347875
Daucus conchitae Greuter	0236	Turkey, Muğla, Marmaris, Datça Pen- insula, northern extremity at Knidos, 36°41′N 27°22′E, 100 m, 8 Apr 2008, *Gardner, Knees, Barker-Mill & Layman 8120* (E 00251858) [specimen labelled as *Daucus bicolor*]		KT347656		KT347780		KT347847

Taxon	Accession ID	Voucher information	GenBank accession numbers					
			ETS	ITS	*rpoB-trnC* spacer	*rpoC1* intron	*rpl16* intron	*rps16* intron
Daucus decipiens (Schrad. & J.C.Wendl.) Spalik, Wojew., Banasiak & Reduron	0613	Portugal, Madeira, Riberio Frio, cult. University of Oslo, the Botanical Garden, seed no. 447, garden no. 257, 12 Mar 1997	*MK142910	KT347683				
	1910 Av	Portugal, Madeira, Folhadal, 20-Oct-2016, *Francisco Fernandes s.n.*, (MADJ 14328)				*MK142825	*MK142853	
	2015 Av	Portugal, Madeira, Funchal, Bot. Garden, May-2017, *Francisco Fernandes s.n.* (WA 0000067159)						
	2017 Ag	Portugal, Madeira, Funchal, Bot. Garden, May-2017, *Francisco Fernandes s.n.* (WA 0000067158)						
	0435							AF123737
Daucus della-cellae (Asch. & Barbey ex E.A.Durand & Barratte) Spalik, Banasiak & Reduron	1728 Av	Libya, Wadi el Kouf, W of Beida, vertical and overhanging cliffs, 300 m, 28 Mar 1970, *Davis 50209* (E 00040973)		AF073565				KT347866
Daucus durieua Lange	0040	Israel, Samarian Desert, near Sartaba, limestone slopes, very dry habitat, 100 m, 21 Apr 1996, *Cohen s.n.* (WA 0000050891)		KT347657		KT347781		KT347848
Daucus edulis (Lowe) Wojew., Reduron, Banasiak & Spalik	0013	Portugal, Madeira, cult. Cons. bot. Mulhouse no. 98141	*MK142906	KT347684		KT347796	*MK142849	KT347865
	2014 Av	Portugal, Madeira, Funchal, Bot. Garden, 28-Apr-2017, *Francisco Fernandes s.n.*, (WA 0000067160)						
	2016 Ag	Portugal, Madeira, Funchal, Bot. Garden, May-2017, *Francisco Fernandes s.n.* (WA 0000067158)						
Daucus elegans (Webb ex Bolle) Spalik, Banasiak & Reduron	0039	Spain, Canary Islands, Tenerife, Cabeza del Tejo, path to El Draguillo, 21 Apr 2009, *P. Danton s.n.* (ORT 46239)	*MK142907	KT347674		KT347791	*MK142850	KT347860

**Taxon**	**Accession ID**	**Voucher information**	**GenBank accession numbers**					
			ETS	ITS	*rpoB-trnC* spacer	*rpoC1* intron	*rpl16* intron	*rps16* intron
	2070 Ag	Spain, Canary Islands, Tenerife, Vueltas de Taganana, *Alfredo Reyes Betancort s.n.* (ORT 46239 & WA 0000067156)						
Daucus glochidiatus (Labill.) Fisch., C.A.Mey. & Avé-Lall.	0455	Australia, North West Plains, between Hesso & Tent Hill, 19 Aug 1968, *Blaylock 938* (KRAM 089717)		FJ415160	KT347740	KT347782		KT347850
Daucus guttatus Sm.	0458	Greece, Crete, Agios Vasileios, 29 Sep 1985, *Risse 2101* (B)		KT347662		KT347784		KT347852
Daucus incognitus (C.Norman) Spalik, Reduron & Banasiak	0046	Kenya, Nairobi, DNA supplied by E. Knox (coll. 2578; WA 0000050886)		KT347648		KT347774		KT347842
	G136						AF094331	
Daucus insularis (Parl. ex Webb) Spalik, Wojew., Banasiak & Reduron	2260	Reading University, Plant Science Laboratories, Fruit & Seed Collection, 1972, 3348 from 4688	*MK142901				*MK142844	*MK142886
	G106	Cult. Botanical Garden, Copenhagen, Denmark, 30 Aug 1982, *Hansen 53* (C) [specimen labelled as *T. hirta* J.A.Schmidt]		KF160739				
	2062 Ag	Cape Verde Islands, Saint Vincente, Mount Verde, NE slope, non-cultivated zone between cornfield and road, 4-Dec-1983, *N. Kilian 763* (FR 0029090)						
Daucus involucratus Sm.	0037	Greece, Crete, Lassithi, near Pachia Ammos, 20 May 1998, *Charpin 25294* (G 467629)		KT347663		KT347785		KT347853
Daucus littoralis Sm.	0008	Israel, Carmel mountain, cult. Cons. bot. Mulhouse no. 99080, *Reduron s.n.* (Reduron, pers. coll.)		KT347666		KT347788		KT347855
	0461	Iran, prov. Mazandaran, Mahmud Ãbãd, Caspian Sea coast (Daryã-ye-Khazar), 15 Jul 2004, *Wojewódzka & Zych s.n.* (WABG)			KT347741			
Daucus mirabilis (Maire & Pamp.) Reduron, Banasiak & Spalik	0244	Libya, Roman aqueduct near Labrag, E of Shahat (Cyrene), 700 m, 29 Mar 1970, *Davis 50249* (E 00025551)		KT347690				

Taxon	Accession ID	Voucher information	GenBank accession numbers					
			ETS	ITS	*rpoB-trnC* spacer	*rpoC1* intron	*rpl16* intron	*rps16* intron
Daucus montanus Humb. & Bonp	0017	Chile, Juan Fernandez archipelago, cult. Cons. bot. Mulhouse no. 98050, *Reduron s.n.* (Reduron, pers. coll.)	*MK142902	KT347668		KT347789	*MK142845	KT347857
Daucus muricatus L.	0271	Algeria, SW side of Bouchegouf (Souk Ahras to Guelma), 200–250 m, 21 May 1975, *Davis 58080* (E 00324297)		KT347676		KT347793		KT347862
Daucus pumilus (L.) Hoffmanns. & Link	0228	Tunisia, Gammarth (N of Tunis), 50-100 m, 26 Apr 1975, *Davis 56740b* (E 00000867)		KT347692				
	G137						AF094329	
	G139							AF123728
Daucus pusillus Michx.	0466	Argentina, prov. de Mendosa, Dep. Malargüe, 20 km S Malargüe, Cerro Chihuido, 18 Jan 1988, *Leuenberger & Arroyo 3867* (B)	*MK142903	KT347673		KT347790	*MK142846	KT347859
Daucus rouyi Spalik & Reduron	2085 Ag	France, Haut-Rhin, Conservatoire Botanique de Mulhouse no. 11-096 , 6-IX-2017, *J.P. Reduron s.n.*	*MK142905			*MK142824		
	0523	France, Corsica, cult. Cons. bot. Mulhouse no. 99143, 9 Sep 2000		FJ415157			*MK142848	KT347868
Daucus setifolius Desf.	0237	Spain, prov. Malaga, Sierra Bermeja, 8 km N of Estepona on road to Jubrique, 36°29′N 5°14′W, 500 m, 28 Aug 1991, *Gardner, Knees & Read 4837* (E 00100819) [specimen labelled as *D. crinitus*]		KT347678		KT347794		
	0467	Algeria, Kherrata, Jun 1896, *Reverchon 234* (B)						KT347864
Daucus syrticus Murb.	0468	Libya, Tripoli, 3 May 1933, *Bornmüller 711* (B)		KT347689		KT347797		KT347867
Daucus tenuisectus Coss. ex Batt.	0266	Morocco, road Asni to Tahnaout, 1250 m, dry slopes by roadside, 31 May 1936, *Balls 2504* (E 00324292)		KT347681		KT347795		
Daucus tenuissimus (A.Chev.) Spalik, Wojew., Banasiak & Reduron	2206 Ag	France, Haut-Rhin, Conservatoire Botanique de Mulhouse no. 12-025 , *J.-P. Reduron,*	*MK142904			*MK142823	*MK142847	*MK142887

Taxon	Accession ID	Voucher information	GenBank accession numbers					
			ETS	ITS	*rpoB-trnC* spacer	*rpoC1* intron	*rpl16* intron	*rps16* intron
	G097	Cape Verde Islands, Montinho, Conservatoire Botanique de Mulhouse no. 2036 *Hildenbrand, Meyer & Reduron* (ILL; Reduron, pers. coll.)		DQ516357				
Ekimia bornmuelleri (Hub.-Mor. & Reese) H.Duman & M.F.Watson	0655	Turkey, Burdur, Yeşilova, S of Salkda Lake, *H. Duman & Karaveliogullari 5071* (E 00064866)		KT347640				KT347810
Ekimia petrophila (Boiss. & Heldr.) Baczyński, Banasiak & Spalik	0509	Turkey, Denizli, Babadağ, 23 Aug 1950, *Davis 18412* (E 00042004)		KT347641		KT347747		KT347811
Laser affine (Ledeb.) Wojew. & Spalik	0480	Georgia, region Akhaltsikhe, 5 km from Lodidzori, 18 July1965, *Shreter & Pimenov 394* (LE)		FJ415151	KT347718	KT347745		KT347808
Laser archangelica (Wulfen) Spalik & Wojew.	0481	Slovenia, Mt. Slivnica above Grahovo near Cerknica Lake, 12 Aug 1967, *Mayer 63416* (KRAM 092698)		FJ415153	KJ832093	KJ832098		KJ832103
Laser carduchorum (Hedge & Lamond) Wojew. & Spalik	0483	Turkey, Bitlis/Van, 10 km SE of Pelli, 8 Jul 1954, *Davis & Polunin 22551* (E 00198181)		FJ415117	KJ832094	KJ832099		KJ832104
Laser stevenii (Fisch., C.A.Mey. & Trautv.) Wojew. & Spalik	0518	Georgia, Lower Svanetia, Lentekhi, near Kakhuri village, 12 May 1979, *Muibaniani & al. s.n.* (LE)		FJ415152	KT347719	KT347746		KT347809
Laser trilobum (L.) Borkh. ex G.Gaertn., B.Mey. & Scherb.	0611	France, environs de Nancy, Jardin botanique de Nancy, cult. Cons. bot. Mulhouse no. 98020B (Reduron, pers. coll.)		KT347638		KT347744		KT347807
	G138						AF094335	
Laserpitium gallicum L. subsp. *gallicum*	0487	France, Minerve, 23 Jul 1979, *Reduron* no. 19790723-13 (Reduron, pers. coll.; WA)		FJ415128	KT347722	KT347749		KT347816
Laserpitium halleri Crantz	0493	France, Alps, col du Lautaret, 3 Aug 1869, *Reverchon s.n.* (KRAM)		FJ415130				
Laserpitium krapfii subsp. *gaudinii* (Moretti) Thell.	0497	Italy, Bergamo Alps, 17 Jul 1969, *Charpin s.n.* (G)		FJ415125	KT347726	KT347753		KT347820
Laserpitium latifolium L.	0498	Poland, Suwałki region, Giby, 8 Aug 1975, *Sudnik s.n.* (WA)		FJ415131		KT347754		KT347822

Taxon	Accession ID	Voucher information	GenBank accession numbers					
			ETS	ITS	*rpoB-trnC* spacer	*rpoC1* intron	*rpl16* intron	*rps16* intron
	2081 Ag	Poland, Suwałki County, near Suchar lake, close to Krzywe Lake, 15-Aug-1974, *Z. Podbielkowski s.n.* (WA 0000050201)						
Laserpitium peucedanoides L.	0510	Montenegro, Kotor, Guslice, 2 Aug 1957, *Zarzycki s.n.* (KRAM 255125)		FJ415133				
Laserocarpum pseudomeum (Orph., Heldr. & Sart. ex. Boiss.) Spalik, Wojew., Constantin. & S. R. Downie	0513	Greece, Mt. Giona near Kaloskopi, 10 Jun 1985, *Gustavsson 9672* (G 458489)		FJ415134		KT347748		KT347812
Orlaya grandiflora (L.) Hoffm.	0258	Slovakia, distr. Levice, Šahy, Studený Vrch, 190 m, 29 May 1968, *Smejkal & Vicherek 1544* (E 00324284)	*MK142909	KT347646		KT347771	*MK142909	KT347840
Siler montanum Crantz	0517	Montenegro, Žabljak, Durmitor, upper slopes of Savin Kuk, 30 Jul 1984, *Gardner & Gardner 2455* (E 00043177)	*MK142911				*MK142854	
	0516	Spain, prov. Burgos, Santo Domingo los Silos, gorge of Rio Yecla, 13 Jul 1980, *Gardner & Gardner 781* (E 00043183)		FJ415112	KT347720			KT347814
	G070	Germany, cult. Johannes Gutenberg University (no. 1112), *Downie 71* (ILL)				U36296		
Silphiodaucus hispidus (M.Bieb.) Spalik, Wojew., Banasiak, Piwczyński & Reduron	0494	Turkey, Kastamonu, Araç, 2 Aug 1962, *Davis, Coode & Yaltirik 38795* (E 00042016)	*MK142900	FJ415154		KT347772	*MK142843	
Silphiodaucus prutenicus (L.) Spalik, Wojew., Banasiak, Piwczyński & Reduron	0649<br>2082 Ag	Poland, Klaudyn (near Pruszków), 2-Aug-1971, *Nowak s.n.* (WA 0000050200)	*MK142908	AF336374		KT347773	*MK142851	KT347841
Thapsia eliasii (Sennen & Pau) Wojew., Banasiak, Reduron & Spalik	0486	Spain, Pias, San Augustin, 11 Aug 1985, *Rico s.n.* (JACA ex SALA 41460)		FJ415120	KT347727	KT347755		KT347824

Taxon	Accession ID	Voucher information	GenBank accession numbers					
			ETS	ITS	*rpoB-trnC* spacer	*rpoC1* intron	*rpl16* intron	*rps16* intron
Thapsia garganica L.	0526	Tunisia, 15 km SE of Korbous, W side of Cap Bon peninsula, 27 Apr 1975, *Davis & Lamond D 56827* (E 00198186)		FJ415144	KT347731	KT347759		KT347828
Thapsia gummifera (Desf.) Spreng.	0520	Portugal, Porto de Lagos, cult. Cons. bot. Mulhouse no. 9309, 9 Sep 2000		FJ415139	KT347733	KT347761		KT347830
Thapsia meoides Guss.	0473	Morocco, WN, N of Oued Cherrat, SW of Rabat, 9 Apr 1972, *Davis 54321* (E 00198184) [specimen labelled as *Eleoselinum asclepium* subsp. *millefolium*]		FJ415138		KT347763		KT347832
Thapsia nestleri (Soy.-Will.) Wojew., Banasiak, Reduron & Spalik	0505	Spain, Huesca, Sallent de Gállego, 22 Aug 2000, *Montserrat s.n.* (JACA R265475)		FJa415121	KT347728	KT347756		KT347825
Thapsia tenuifolia Lag.	0469	Portugal, cult. Cons. bot. Mulhouse no. 9372, 7 Sep 2000		FJ415140	KT347735	KT347766		KT347835
Thapsia thapsioides (Desf.) Simonsen, Rønsted, Weitzel & Spalik	0477	Tunisia, N, Ain Sebaa to Jebbara beach (E of Tabarka), 12 May 1975, *Davis & Lamond D 57768* (E 00040997)		FJ415141	KT347736	KT347767		KT347836
Thapsia transtagana Brot.	0524	Morocco, N of Tamri, 19 Mar 1969, *Davis & Davis D 48431* (E 00198188) [specimen labelled as *Thapsia garganica*]		FJ415146	KT347738	KT347769		KT347838
Thapsia villosa L.	0528	Morocco, 16 km E of Tafraout on road to Ait Baha, climbing up to pass at E end of Amelm valley, 14 May 1982, *Davis & King D 68336* (E 00198189)		FJ415147	KT347739	KT347770		KT347839

Thiers, B. M. 2013 onward (continously updated) Index Herbariorum: A global directory of public herbaria and associated staff. New York Botanical Garden's Virtual Herbarium. Website http://sweetgum.nybg.org/ih/ [accessed 31.12.2019].
