# Supplementary material for: Parallel evolution of arborescent carrots (Daucus) in Macaronesia
Source: Am J Bot. 2020 Mar 8;107(3):394–412. doi: 10.1002/ajb2.1444 (PMC7155066; doi:10.1002/ajb2.1444)
Supplement: Supplementary file 2 — APPENDIX S2. Characteristics of the data set used in phylogenetic analysis table. [file AJB2-107-394-s002.pdf]

Appendix S2 Characteristics of the datasets used in phylogenetic analysis. All numbers are for matrices with ambiguously aligned positions removed using trimAl.

Data sets	ETS	ITS	**nrDNA combined**	*rpoB-trnC* spacer	*rpoC1* intron	*rpl16* intron	*rps16* intron	**pDNA combined**	**All combined**
Sequence length variation [bp]	454–456	212–592	212–1048	1131–1256	555–726	501–574	797–831	725–3387	587–4435
Number of unambiguously aligned positions:									
Total	456	592	1048	1259	726	574	831	3390	4438
Constant	268	254	522	1023	600	488	688	2799	3321
Autapomorphic	69	104	173	145	79	52	87	363	536
Parsimony informative	119	234	353	91	47	34	56	228	581
Containing gaps	5	39	44	182	24	47	31	284	328
Percentage of gaps/missing data	76.69%	1.2%	34.05%	66.4%	20.45%	70.51%	8.54%	43.07%	40.94%
Mean indel length overall [bp]	1.75	1.81	1.8	2.75	1.29	5.06	2.05	2.71	2.6
Maximum indel length overall [bp]	2	9	9	24	5	12	8	24	24
