# Supplementary material for: Parallel evolution of arborescent carrots (Daucus) in Macaronesia
Source: Am J Bot. 2020 Mar 8;107(3):394–412. doi: 10.1002/ajb2.1444 (PMC7155066; doi:10.1002/ajb2.1444)
Supplement: Supplementary file 3 — APPENDIX S3. ITS and plastid markers median clock rate values. [file AJB2-107-394-s003.pdf]

Appendix S3 ITS and plastid markers median clock rate values

A ITS median clock rate values [number of expected substitutions/site/million of years] x 10^{-3}. Branch thickness is proportional to the corresponding numerical value. Mostly Monocarpic Clade (MMC) lineages show, on average, twice as fast the rate of ITS molecular evolution as Perennial Polycarpic Grade (PPG).

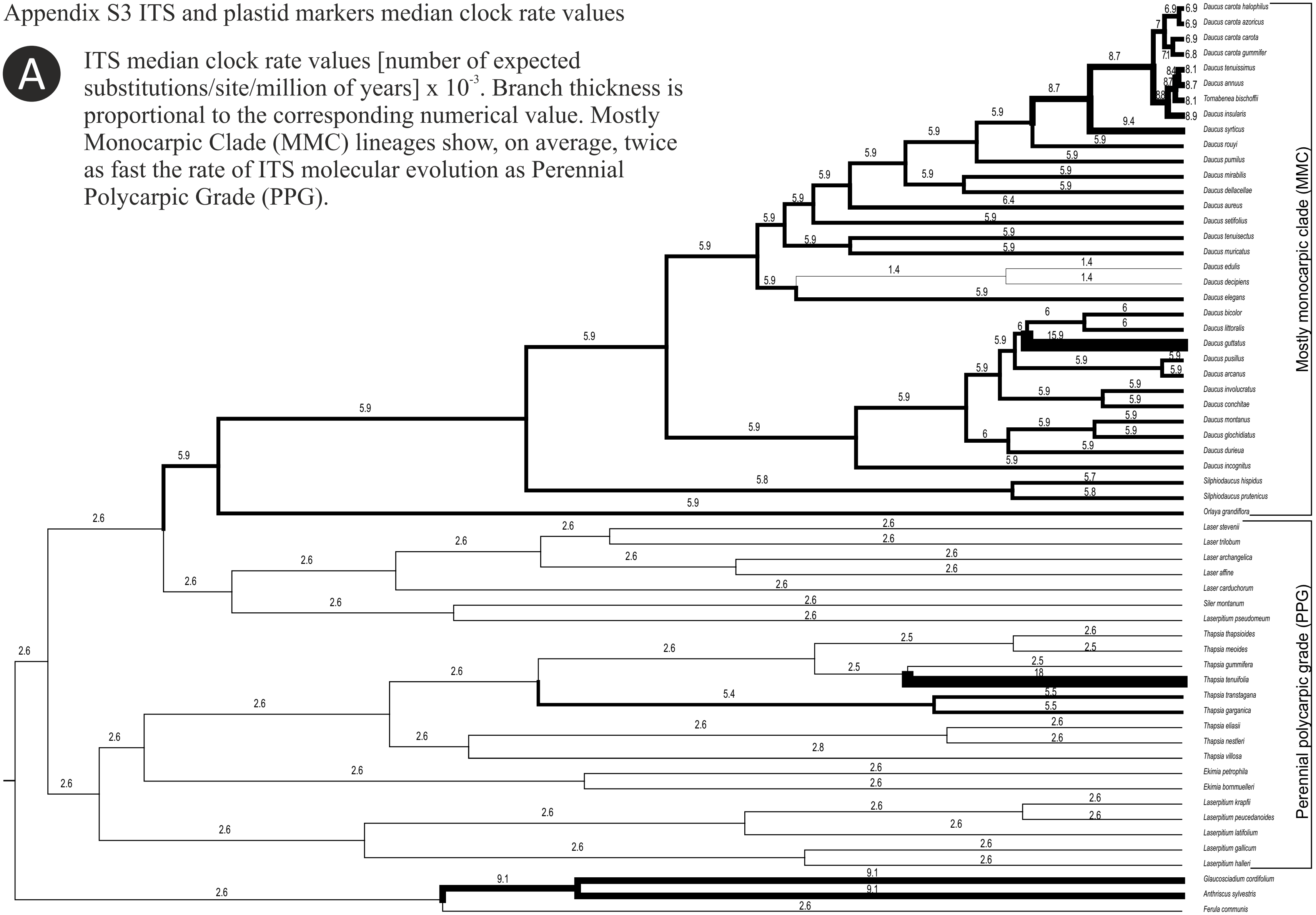

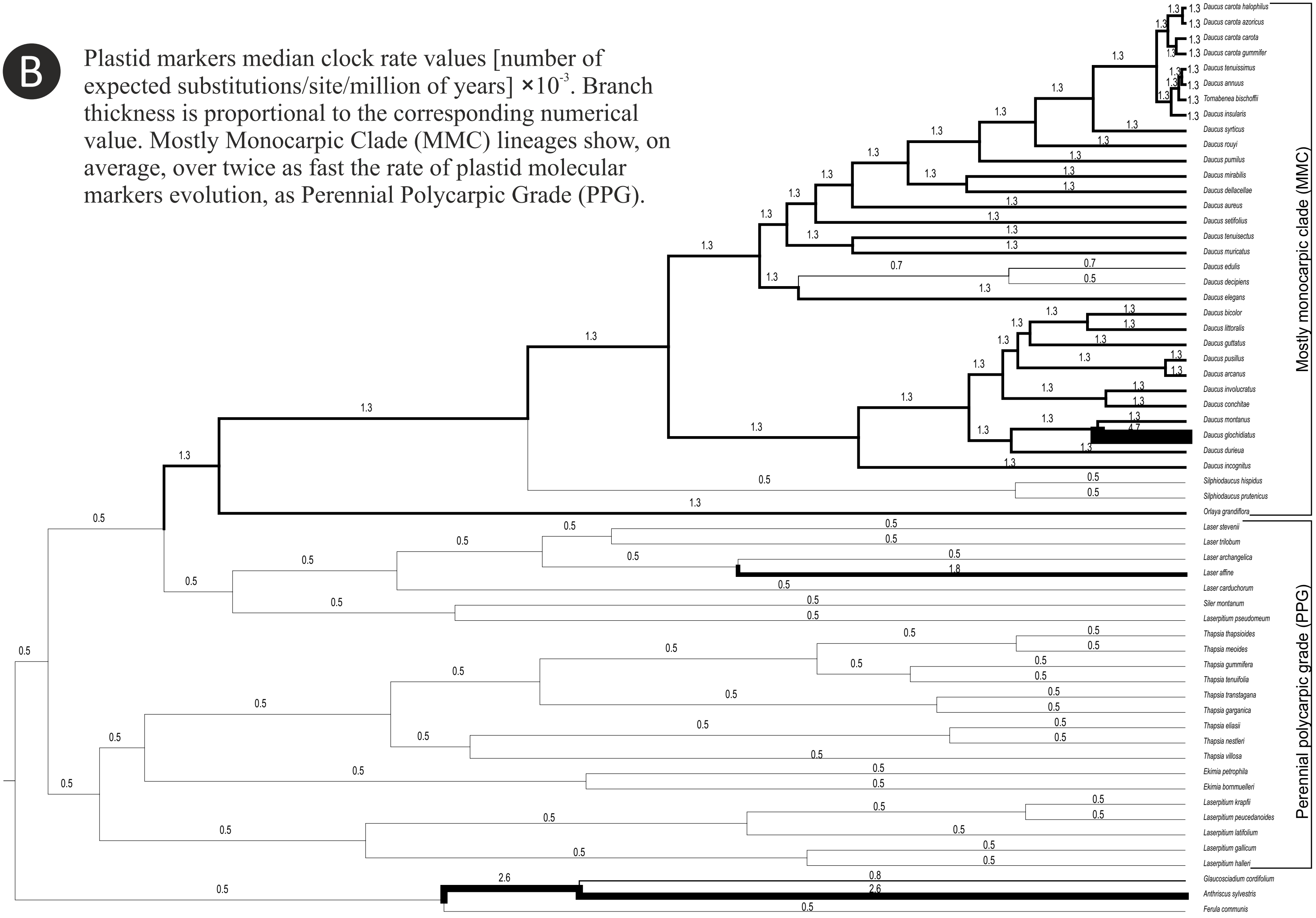

Plastid markers median clock rate values [number of expected substitutions/site/million of years] $\times 10^{-3}$. Branch thickness is proportional to the corresponding numerical value. Mostly Monocarpic Clade (MMC) lineages show, on average, over twice as fast the rate of plastid molecular markers evolution, as Perennial Polycarpic Grade (PPG).
