# Supplementary material for: Parallel evolution of arborescent carrots (Daucus) in Macaronesia
Source: Am J Bot. 2020 Mar 8;107(3):394–412. doi: 10.1002/ajb2.1444 (PMC7155066; doi:10.1002/ajb2.1444)
Supplement: Supplementary file 4 — APPENDIX S4. Wood trait measurements. [file AJB2-107-394-s004.pdf]

Appendix S4 Table with measurements of wood traits following guidelines of IAWA Committee (1989). Measurements that could not have been taken for technical reasons are marked with a dash (-), while those that were not measured for biological reasons (e.g., lack of a given trait) are given explanations instead.

**Taxon**	**Specimen ID**	**Length of vessel elements min**<br>**minLVE [µm]**	**Length of vessel elements mean**<br>**meanLVE [µm]**	**Length of vessel elements max**<br>**maxLVE [µm]**	**Length of fibers min**<br>**minLF [µm]**	**Length of fibers mean**<br>**meanLF [µm]**	**Length of fibers max**<br>**maxLF [µm]**
Daucus della-cellae	1728	73	143	276	122	207	347
Daucus della-cellae [inner]	1728	-	-	-	Wood without fibers	Wood without fibers	Wood without fibers
Daucus della-cellae [outer]	1728	-	-	-	122	207	347
Daucus edulis [parenchymatous]	2014	162	227	312	Wood without fibers	Wood without fibers	Wood without fibers
Daucus edulis [fibrous]	2016	170	274	555	211	407	631
Laserpitium latifolium	2081	82	264	638	195	414	940

Laserpitium latifolium [parenchymatous]	2081	-	-	-	Wood without fibers	Wood without fibers	Wood without fibers
Laserpitium latifolium [fibrous]	2081	-	-	-	195	414	940
Silphiodaucus prutenicus	2082	114	267	569	183	415	869
Silphiodaucus prutenicus [parenchymatous]	2082	-	-	-	Wood without fibers	Wood without fibers	Wood without fibers
Silphiodaucus prutenicus [fibrous]	2082	-	-	-	183	415	869
Daucus bischoffii	2060	131	285	508	201	373	562
Daucus carota	2057	133	330	743	168	467	1163
Daucus decipiens	2015	272	481	702	194	530	1168
Daucus elegans	2070	143	311	488	174	386	688
Daucus insularis	2062	130	245	330	109	276	330
Daucus rouyi	2085	89	216	459	129	247	412
Daucus tenuissimus	2354	109	221	336	227	367	526

Taxon	Specimen ID	No. of vessel lumina per sq.mm mean<br>NVS [N/sq mm]	No. of vessel lumina per group mean<br>NVG [N/group]	No. of vessel lumina per group max<br>maxNVG [N/group]	Thickness of fiber walls min<br>minFWT [μm]	Thickness of fiber walls mean<br>meanFWT [μm]	Thickeness of fibre walls max<br>maxFWT [μm]
Daucus della-cellae	1728	321	3.6	8	1.14	2.05	4.01
Daucus della-cellae [inner]	1728	206	4.3	16	Wood without fibers	Wood without fibers	Wood without fibers
Daucus della-cellae [outer]	1728	477	3	8	1.14	2.05	4.01
Daucus edulis [parenchymatous]	2014	16	2.7	5	Wood without fibers	Wood without fibers	Wood without fibers
Daucus edulis [fibrous]	2016	22	3.9	12	0.87	1.58	2.52
Laserpitium latifolium	2081	114	3.4	6	1.4	2.1	2.8
Laserpitium latifolium [parenchymatous]	2081	124	5.8	18	Wood without fibers	Wood without fibers	Wood without fibers
Laserpitium latifolium [fibrous]	2081	34	3.8	8	1.4	2.1	2.8

Silphiodaucus prutenicus	2082	77	2.4	13	1.8	2.9	4.1
Silphiodaucus prutenicus [parenchymatous]	2082	64	4.1	10	Wood without fibers	Wood without fibers	Wood without fibers
Silphiodaucus prutenicus [fibrous]	2082	86	3.4	7	1.8	2.9	4.1
Daucus bischoffii	2060	159	3.7	10	1.5	2.1	4.3
Daucus carota	2057	176	4.3	8	1.2	2.2	3.2
Daucus decipiens	2015	72	3.2	12	1.1	2	3
Daucus elegans	2070	74	3.1	5	0.9	1.6	2.3
Daucus insularis	2062	48	3.5	11	1.8	2.8	3.5
Daucus rouyi	2085	35	3.3	12	1.9	2.8	3.6
Daucus tenuissimus	2354	214	10.5	23	1.4	2.5	3.7

Taxon	Specimen ID	Thickness of vessel wall min<br><br>minVWT [µm]	Thickness of vessel wall mean<br><br>meanVWT [µm]	Thickeness of vessel walls max<br><br>maxVWT [µm]	Tang diameter of vessels min<br><br>minDV [µm]	Tang diameter of vessels mean<br><br>meanDV [µm]	Tang diameter of vessels max<br><br>maxDV [µm]
Daucus della-cellae	1728	1.1	2.3	3.8	4.25	15.6	33.34
Daucus della-cellae [inner]	1728	0.96	1.6	2.35	4.96	9.8	18.64
Daucus della-cellae [outer]	1728	1.07	2.24	3.27	6.5	18.3	34.3
Daucus edulis [parenchymatous]	2014	2.7	5	9.4	24.7	57.2	82
Daucus edulis [fibrous]	2016	1.8	3.3	5.9	20	45	70
Laserpitium latifolium	2081	1.6	3.4	5.5	9.1	27.9	49.9
Laserpitium latifolium [parenchymatous]	2081	2.5	4.3	6.9	10.9	29.4	55
Laserpitium latifolium [fibrous]	2081	2.3	4	6.5	9.6	26.1	50.8

Silphiodaucus prutenicus	2082	1.4	2.6	4.4	15.7	26.8	52
Silphiodaucus prutenicus [parenchymatous]	2082	2.2	3.2	4.7	7.8	20.8	41
Silphiodaucus prutenicus [fibrous]	2082	1.3	2.7	4.7	7.5	21.6	62
Daucus bischoffii	2060	1.3	2.6	3.9	15.5	24.3	43
Daucus carota	2057	1.4	2.4	3.4	7.6	11.8	21.1
Daucus decipiens	2015	1.6	2.6	3.9	7.8	23.4	44.7
Daucus elegans	2070	1.2	1.9	2.7	9	24.1	35.4
Daucus insularis	2062	1.6	3.1	4.6	14.6	38.7	69.6
Daucus rouyi	2085	2.3	4.5	6.8	13.8	24.7	41.3
Daucus tenuissimus	2354	1.2	3	4.6	6.3	23.32	45

Taxon	Specimen ID	Percent of solitary vessels<br><br>PSV [%]	Width of multiseriate rays in cells mean<br><br>meanRN [N of cells]	Width of multiseriate rays in cells max<br><br>maxRN [N of cells]	Height of multiseriate rays um min<br><br>minHR [µm]	Height of multiseriate rays um mean<br><br>meanHR [µm]	Height of multiseriate rays um max<br><br>maxHR [µm]
Daucus della-cellae	1728	25	3.97	6	486.6	658.1	891.8
Daucus della-cellae [inner]	1728	28	No difference along transect	No difference along transect	No difference along transect	No difference along transect	No difference along transect
Daucus della-cellae [outer]	1728	37	No difference along transect	No difference along transect	No difference along transect	No difference along transect	No difference along transect
Daucus edulis [parenchymatous]	2014	41	4	7	376.3	1026.3	2324.2
Daucus edulis [fibrous]	2016	27	3.9	7	233.7	862.7	4089
Laserpitium latifolium	2081	10	4.2	9	169.3	725	2351
Laserpitium latifolium [parenchymatous]	2081	16	-	-	-	-	-
Laserpitium latifolium [fibrous]	2081	26	4.3	9	83.5	259.2	785

Silphiodaucus prutenicus	2082	41	5.5	14	234.3	690.3	1745.8
Silphiodaucus prutenicus [parenchymatous]	2082	20	4.9	9	-	-	-
Silphiodaucus prutenicus [fibrous]	2082	35	4.7	9	191.8	470.4	743.6
Daucus bischoffii	2060	35	3.26	9	129.7	688.6	1468.8
Daucus carota	2057	31	3.5	5	2000	-	-
Daucus decipiens	2015	44	3.8	15	91.4	341.8	1082.8
Daucus elegans	2070	48	Wood rayless	Wood rayless	Wood rayless	Wood rayless	Wood rayless
Daucus insularis	2062	14	3.4	5	95.8	519.6	1957.6
Daucus rouyi	2085	55	6	9	4000	-	-
Daucus tenuissimus	2354	17	2.8	5	200.3	472.7	1856.7

Taxon	Specimen ID	No. of multiseriate rays per 1 mm<br><br>meanNMR	No. of uniseriate rays per 1 mm<br><br>meanNUR	Total no. of rays per 1 mm<br><br>NTR	Diameter (horizontal) of intervessel pits min<br><br>minDIP [μm]	Diameter (horizontal) of intervessel pits mean<br><br>meanDIP [μm]	Diameter (horizontal) of intervessel pits max<br><br>maxDIP [μm]
Daucus della-cellae	1728	1.5	1	2.5	6.12	10.58	13.5
Daucus della-cellae [inner]	1728	No difference along transect	No difference along transect	No difference along transect	No difference along transect	No difference along transect	No difference along transect
Daucus della-cellae [outer]	1728	No difference along transect	No difference along transect	No difference along transect	No difference along transect	No difference along transect	No difference along transect
Daucus edulis [parenchymatous]	2014	6.2	1.2	7.4	8.2	15.3	25.4
Daucus edulis [fibrous]	2016	2.9	0.7	3.6	6	12.6	33
Laserpitium latifolium	2081	3.4	1.9	5.3	4.3	7	10.9
Laserpitium latifolium [parenchymatous]	2081	-	-	-	5.5	10.9	20.7
Laserpitium latifolium [fibrous]	2081	3.4	1.9	5.3	4.9	12.7	28.5

Silphiodaucus prutenicus	2082	3.8	2.1	5.9	4.1	9.2	20.9
Silphiodaucus prutenicus [parenchymatous]	2082	-	-	-	4.2	7.4	17
Silphiodaucus prutenicus [fibrous]	2082	3.8	2.1	5.9	3.7	7.5	18.2
Daucus bischoffii	2060	2.67	3.33	6	5.9	7.7	10.7
Daucus carota	2057	3.8	1.4	5.2	4.3	5.6	8.1
Daucus decipiens	2015	4.4	1.4	5.8	7.7	12.9	32.3
Daucus elegans	2070	Wood rayless	Wood rayless	Wood rayless	4.9	6.2	8.3
Daucus insularis	2062	3.9	1.4	5.3	5	7.4	10.3
Daucus rouyi	2085	3.9	1.5	5.4	4.5	8.2	21.6
Daucus tenuissimus	2354	4.8	0.2	5	3.5	5.1	7.5

Taxon	Specimen ID	Vertical diameter of intervessel pits min<br><br>minVIP [µm]	Vertical diameter of intervessel pits mean<br><br>meanVIP [µm]	Vertical diameter of intervessel pits max<br><br>maxVIP [µm]	LF/LVE<br><br>LF/LVE	Vulnerability index<br><br>V [µm × sq mm]	Mesomorphy index<br><br>M [sq mm]
Daucus della-cellae	1728	1.81	2.48	3.54	1.44	0.05	6.9
Daucus della-cellae [inner]	1728	No difference along transect	No difference along transect	No difference along transect	-	0.05	-
Daucus della-cellae [outer]	1728	No difference along transect	No difference along transect	No difference along transect	-	0.04	-
Daucus edulis [parenchymatous]	2014	2.2	4.4	6.8	-	3.62	823.2
Daucus edulis [fibrous]	2016	3.8	6	7.8	1.49	2.05	559.8
Laserpitium latifolium	2081	2.7	4.6	6.7	1.57	0.25	64.7
Laserpitium latifolium [parenchymatous]	2081	3.5	5	6.8	-	0.24	-
Laserpitium latifolium [fibrous]	2081	3	4.7	5.9	-	0.77	-
Silphiodaucus prutenicus	2082	1.8	3.4	6.3	1.56	0.35	92.6

Silphiodaucus prutenicus [parenchymatous]	2082	3	3.8	5.2	-	0.33	-
Silphiodaucus prutenicus [fibrous]	2082	2.9	4	4.9	-	0.25	-
Daucus bischoffii	2060	4.4	5.9	7	1.31	0.15	43.6
Daucus carota	2057	3.1	4.5	6.1	1.41	0.07	22.1
Daucus decipiens	2015	3.8	5.6	7	1.10	0.32	156.0
Daucus elegans	2070	3.4	4.2	5.8	1.24	0.33	101.4
Daucus insularis	2062	3.1	4.4	6.1	1.12	0.80	196.1
Daucus rouyi	2085	3.5	4.6	6.7	1.15	0.71	152.7
Daucus tenuissimus	2354	3.2	4.4	6.2	-	0.11	24.1

IAWA Committee. 1989. IAWA list of microscopic features for hardwood identification with an appendix on non-anatomial information. *IAWA Bulletin n.s* 10: 219–332.
