# Supplementary material for: Parallel evolution of arborescent carrots (Daucus) in Macaronesia
Source: Am J Bot. 2020 Mar 8;107(3):394–412. doi: 10.1002/ajb2.1444 (PMC7155066; doi:10.1002/ajb2.1444)

Appendix S5 Length-on-age curve for *Daucus decipiens* showing change in vessel element length with growing distance from the pith. Shaded area marks 95% confidence interval, and violin plots with particular measurements marked as dots are in the background. Series “a” and “b” denote measurements taken to the left and right from the pith.

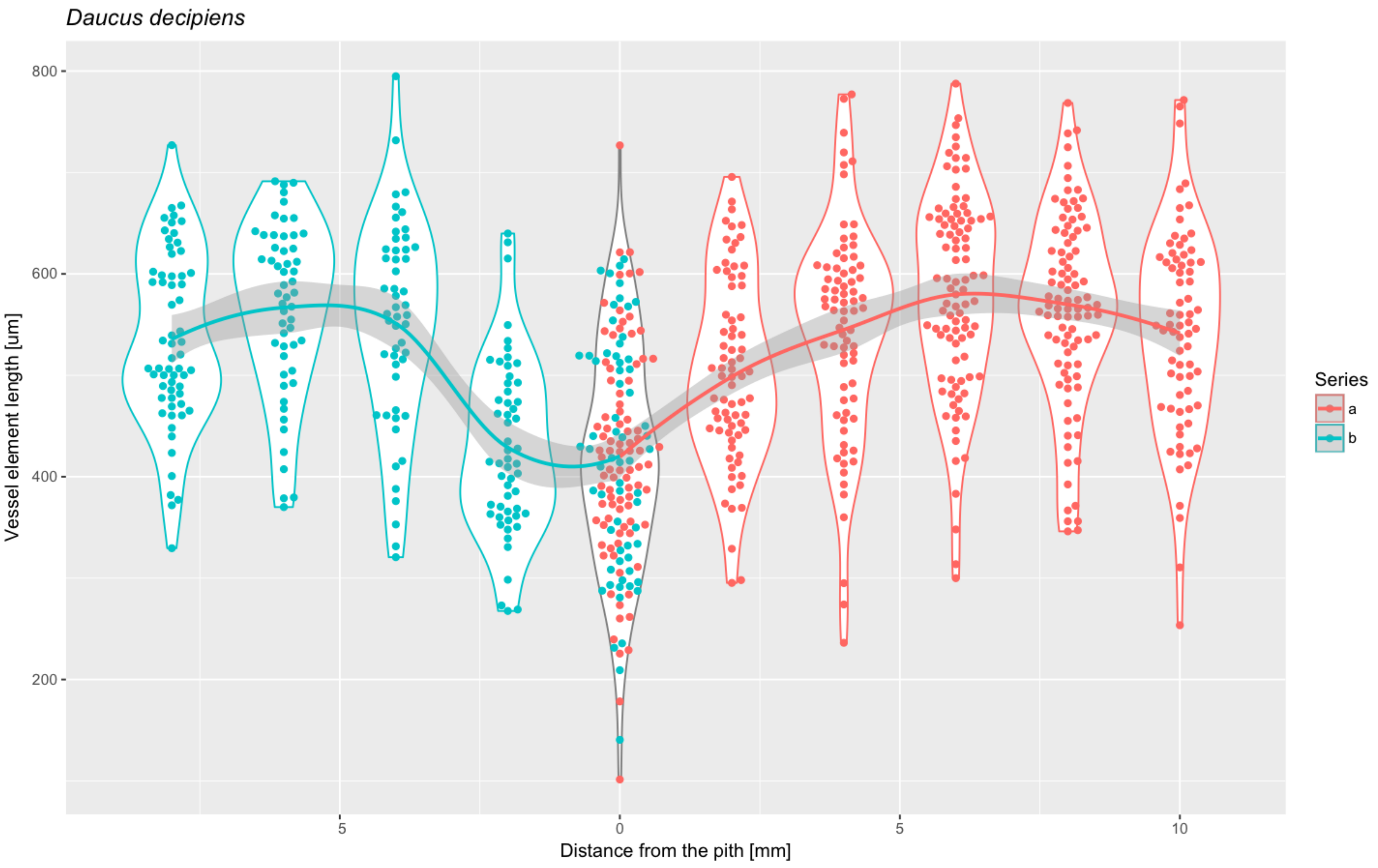

Supplement: Supplementary file 5 — APPENDIX S5. Daucus decipiens length‐on‐age curve. [file AJB2-107-394-s005.pdf]
