# Supplementary material for: Parallel evolution of arborescent carrots (Daucus) in Macaronesia
Source: Am J Bot. 2020 Mar 8;107(3):394–412. doi: 10.1002/ajb2.1444 (PMC7155066; doi:10.1002/ajb2.1444)

Appendix S6 Results of ancestral state estimation obtained with maximum parsimony (branch colors) and maximum likelihood (pie charts). Time scales are in millions of years. Best-fitting models of evolution were: all rates different for growth rings and reproductive strategy, and equal rates/symmetrical for wood porosity and libriform fibers.

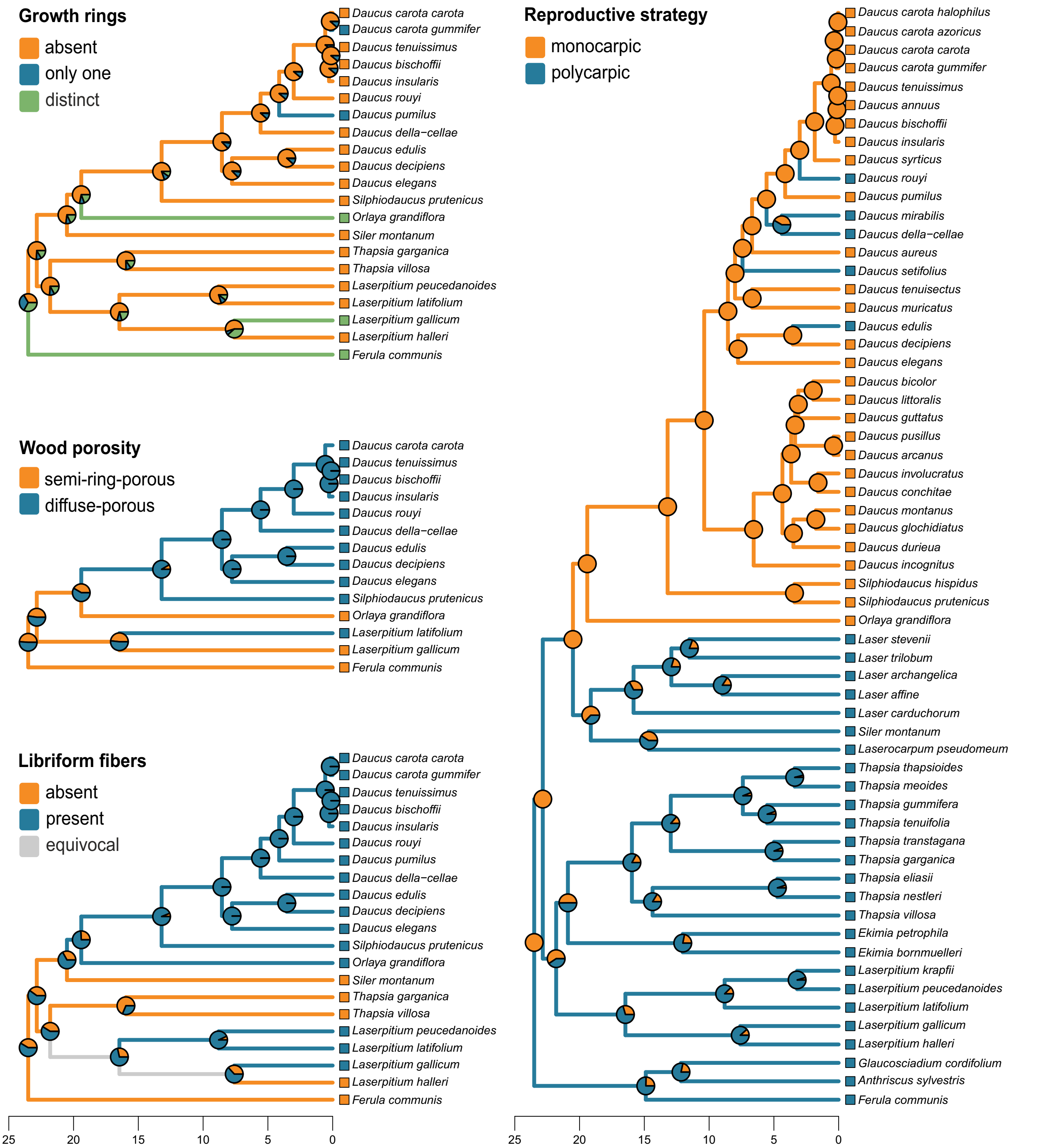

Supplement: Supplementary file 6 — APPENDIX S6. Results of ancestral state estimation for growth rings, wood porosity, libriform fibers, and reproductive strategy. [file AJB2-107-394-s006.pdf]
